# Quantitative 3D real-space analysis of Laves phase supraparticles

**DOI:** 10.1038/s41467-021-24227-0

**Published:** 2021-06-25

**Authors:** Da Wang, Ernest B. van der Wee, Daniele Zanaga, Thomas Altantzis, Yaoting Wu, Tonnishtha Dasgupta, Marjolein Dijkstra, Christopher B. Murray, Sara Bals, Alfons van Blaaderen

**Affiliations:** 1 Soft Condensed Matter, Debye Institute for Nanomaterials Science, Utrecht University, Utrecht, The Netherlands; 2 Electron Microscopy for Materials Science (EMAT), University of Antwerp, Antwerp, Belgium; 3 Department of Chemistry, University of Pennsylvania, Philadelphia, PA United States; 4 Department of Materials Science and Engineering, University of Pennsylvania, Philadelphia, PA United States; 5 NANOlab Center of Excellence, University of Antwerp, Antwerp, Belgium; 6 Present Address: Electron Microscopy for Materials Science (EMAT), University of Antwerp, Antwerp, Belgium; 7 Present Address: Department of Imaging Physics, Delft University of Technology, Delft, The Netherlands; 8 Present Address: Vlaamse Instelling voor Technologisch Onderzoek (VITO), Mol, Belgium

**Keywords:** Nanoparticles, Colloids, Self-assembly, Transmission electron microscopy

## Abstract

Assembling binary mixtures of nanoparticles into crystals, gives rise to collective properties depending on the crystal structure and the individual properties of both species. However, quantitative 3D real-space analysis of binary colloidal crystals with a thickness of more than 10 layers of particles has rarely been performed. Here we demonstrate that an excess of one species in the binary nanoparticle mixture suppresses the formation of icosahedral order in the self-assembly in droplets, allowing the study of bulk-like binary crystal structures with a spherical morphology also called supraparticles. As example of the approach, we show single-particle level analysis of over 50 layers of Laves phase binary crystals of hard-sphere-like nanoparticles using electron tomography. We observe a crystalline lattice composed of a random mixture of the Laves phases. The number ratio of the binary species in the crystal lattice matches that of a perfect Laves crystal. Our methodology can be applied to study the structure of a broad range of binary crystals, giving insights into the structure formation mechanisms and structure-property relations of nanomaterials.

## Abstract (web summary)

3D real-space analysis of thick nanoparticle crystals is non-trivial. Here, the authors demonstrate the structural analysis of a bulk-like Laves phase by imaging an off-stoichiometric binary mixture of hard-sphere-like nanoparticles in spherical confinement by electron tomography, enabling defect analysis on the single-particle level.

## Introduction

Structuring matter by self-assembly (SA) of either nanoparticles (NPs) or micron-sized colloids has progressed significantly over the last decades^1–6^. Compared with single-component assemblies, the fundamental advantage of assembling binary components into a more complex structure is the possibility of creating materials with novel enhanced collective properties (e.g., conductivity) that are distinct from the sum of the two components. The properties can be tailored through not only the size and shape of the individual building blocks but also through the interactions between the binary components and the structure of the assembly^2,7,8^. As the properties of the materials (e.g., conductivity) strongly depend on the presence of defects, which at finite temperatures are commonly present^9^, it is crucial to study the assemblies on a single-particle level.

Laves phases, crystal structures with an *LS*_2_ stoichiometry where the small (*S*) species and the large (*L*) species have a size ratio *γ* = *σ*_*S*_/*σ*_*L*_ with *σ*_*L*(*S*)_ the diameter of the *L*(*S*) species ~0.82, was first found in intermetallic compounds: MgZn_2_, MgCu_2_ and MgNi_2_^10^. Stacking faults have been observed in intermetallic alloys upon synchroshear or aging^11,12^. Excess atoms originating from off-stoichiometry have been found to be accommodated by planar defects, forming for instance a *μ* phase (or structural motifs from a *μ* phase) and Laves-related phases (e.g., *LS*_3_ and *L*_4_*S*_7_)^11^. Moreover, it was found that such defects in off-stoichiometric Laves phase can be thermodynamically more favorable than the formation of a basal synchroshear-induced stacking fault defect structure^11^.

For binary hard spheres (HSs) at size ratios in the range of 0.76 ≤ *γ* ≤ 0.84, the MgZn_2_ structure is the thermodynamically stable structure, although the free-energy difference between the three Laves structures is small (~10^−3^*k*_*B*_*T* per particle)^13,14^. Crystals composed of particles interacting with predominantly hard interactions are prone to form stacking faults, as has been found in the random stacking of a face-centered cubic (FCC) crystal of HS-like particles^15,16^. For this reason, one expects to find stacking faults in Laves phase crystals composed of HS-like particles and in a higher abundance than in intermetallic Laves phases^11,12^, as the free-energy penalty to form stacking faults in single-species crystals is four orders of magnitude higher owing to the attractions present in these atomic systems^17^. The only study we are aware of where the random stacking of Laves phases has been reported is a recent investigation of Laves phases composed of micron-sized microgel colloidal particles using static light scattering^18^.

Analyzing the local structure at for instance defects in crystals is non-trivial with techniques such as (small-angle) X-ray diffraction and electron crystallography, as these usually strongly bias for regularity in the structure^19^. Transmission electron microscopy (TEM) has been used to study the defects in binary nanocrystal superlattices (BNSLs) with a limited thickness of 3–5 unit cells^20^. But as these BNSLs are grown on a liquid–air interface or on top of a flat surface, the crystal orientation is limited to a fixed crystallographic orientation^2^. This limits the observation of the necessary projections to determine the stacking faults. Moreover, defects such as point defects can only be analyzed accurately by information on the single-particle level. Within the last decade, electron tomography has enabled the quantitative study of the real-space structure of NP assemblies^19,21,22^ and even the atomic structure inside a single NP^23–25^ and grain boundaries of nanometals^26^. The quantitative study is not only instrumental to identify defects, but it is also essential to unambiguously identify the correct crystal structure^19^. Moreover, it enables a better understanding of the structural formation mechanisms^27^. It is, however, challenging to perform electron tomography on BNSLs, as at high tilt angles they are no longer electron transparent. Finally, electron tomography is often performed on BNSLs with a limited sample thickness (< 10 layers)^19,28–30^, making the effect of the boundaries of the assemblies possibly quite important on the structure and properties of the assemblies.

This challenge can be tackled by studying a structure with a spherical geometry, which has the advantage that the path length for the electron beam becomes independent of the tilt angle. Electron tomography can be used to study the structure of supraparticles (SPs)^2,31–33^: particles made from particles. These SPs are assembled by drying emulsion droplets containing colloidal particles, resulting in spherical assemblies^2,31–33^. The reconstruction of electron tomograms has enabled the quantitative real-space analysis in 3D of binary SPs^27^. Although the SPs enable the detailed study of the structure of assemblies on the single-particle level, it should be noted, however, that the spherical confinement can also give rise to a structural change to icosahedral order^27,34–38^, which differs from the bulk-like structures of the building blocks. Therefore, it is currently not possible to study the 3D real-space structure of (bulk-like) binary crystals on the single-particle level in a quantitative manner.

Here, we demonstrate by experiments that binary SPs with an excess of small species allow the real-space study of bulk-like binary crystals on the single-particle level, as the effect of the spherical confinement on the structure is negated, suppressing icosahedral symmetry. We prepare core-shell SPs composed of a binary mixture of PbSe (*L*) and CdSe (*S*) nanocrystals (NCs) with a size ratio of 0.78 in an excess of S species (i.e., *N*_*L*_/*N*_*S*_ < 0.5, where *N*_*L*_ and *N*_*S*_ denote the number of the *L* and *S* species, respectively), where the cores are composed of a binary Laves phase and the shells are composed of a crystalline layer of the excess *S* species. We investigate the local symmetry of the particles in real space by advanced electron tomography^27,39^, successfully identifying both species, which enables us to perform a quantitative bond-orientational order parameter (BOP)^40^ analysis. We find that the structure of our SPs is composed of a mixture of the Laves phases, similar to the random stacking of hexagonally-close-packed layers in single-species HSs^15^. Moreover, we find that the number ratio of the binary species in the crystalline core matches the stoichiometry of a perfect Laves phase. We foresee that the use of off-stoichiometric SA of binary crystals in the perfect spherical geometry of SPs will enable quantitative 3D real-space analysis on the single-particle level of bulk-like binary crystals, thus making it possible to more directly correlate the structure and properties of binary crystals in general.

## Results

### The Laves phases

**Fig. 1:**
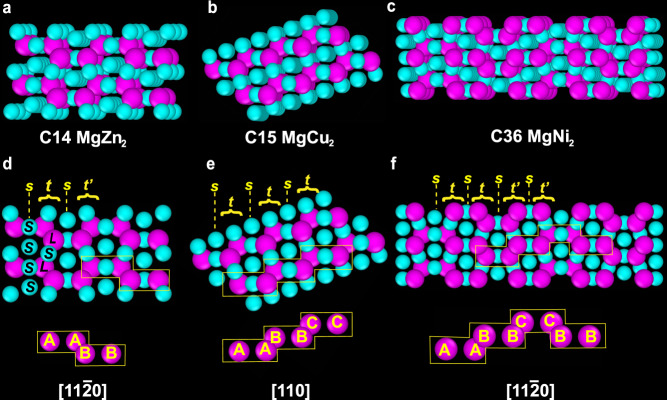
Binary Laves crystal structures. 3D views of the (**a**) C14 (MgZn_2_), (**b**) C15 (MgCu_2_) and (**c**) C36 (MgNi_2_) structures. (**d**) C14 structure viewed along the [11$\bar{2}$0] projection. (**e**) C15 structure viewed along the [110] projection. **f** C36 structure viewed along the [11$\bar{2}$0] projection. For interactive 3D views of the three Laves phases, see Supplementary Data 1–3.

Laves phases (*LS*_2_) are three closely related structures, composed of *S* and *L* spheres: the hexagonal C14, cubic C15 and hexagonal C36 structures (Fig. 1a–c; Supplementary Data 1–3), as found for the intermetallic compounds MgZn_2_, MgCu_2_ and MgNi_2_, respectively^11,41,42^. For the C14 and C15 structures, the packing of the *L* spheres corresponds to hexagonal diamond and cubic diamond structures, respectively, whereas for the C36 structure the *L* spheres occupy the sites of the 4H SiC structure^43,44^. In the C14 and C15 structures, the *S* spheres pack like tetrahedra around the *L* spheres. In the C15 structure, the tetrahedra are jointed point-to-point throughout the structure (forming a pyrochlore structure^45^), whereas in the C14 structure they alternate between base-to-base and point-to-point^46^. In the C36 structure, the *S* spheres pack in tetrahedra where some points are shared, resulting in a structure that locally resembles both C14 and C15^46^. The three Laves structures can be distinguished when viewing along the [11$\bar{2}$0] or [110] projections of the hexagonal or cubic structures, respectively (Fig. 1d–f). Along with these projections, sets of four lines of particles can be identified, consisting of a single line *s* of *S* spheres and a triple-line stack (*t* or *t’*), which contains two lines of *L* spheres separated by a line of *S* spheres (e.g., Fig. 1d). The two forms of the triple-line stacks *t* and *t’*, are mirror reflections of each other with respect to the single line *s* of *S* spheres. The C14, C15 and C36 structures differ in the stacking order of the *s*, *t* and *t’* lines, namely, the C14, C15 and C36 structures exhibit repeated ...*s t s t*’..., ...*s t*... and ...*s t s t s t’ s t’*... stacking sequences, respectively (Fig. 1d–f)^47^. Another way to identify the three Laves structure is based on the stacking of the *L* sphere-dimers. The stacking of the *L* sphere-dimers in the C14, C15 and C36 structures is ...*AABB*..., ...*AABBCC*... and ...*AABBCCBB*..., respectively, when viewing along the [11$\bar{2}$0] or [110] projections of the hexagonal or cubic structure^13^. Hereafter, we will refer to the different structures by the name of their most common intermetallic compounds: MgZn_2_ (C14), MgCu_2_ (C15) and MgNi_2_ (C36).

### SA of binary NCs in bulk

**Fig. 2:**
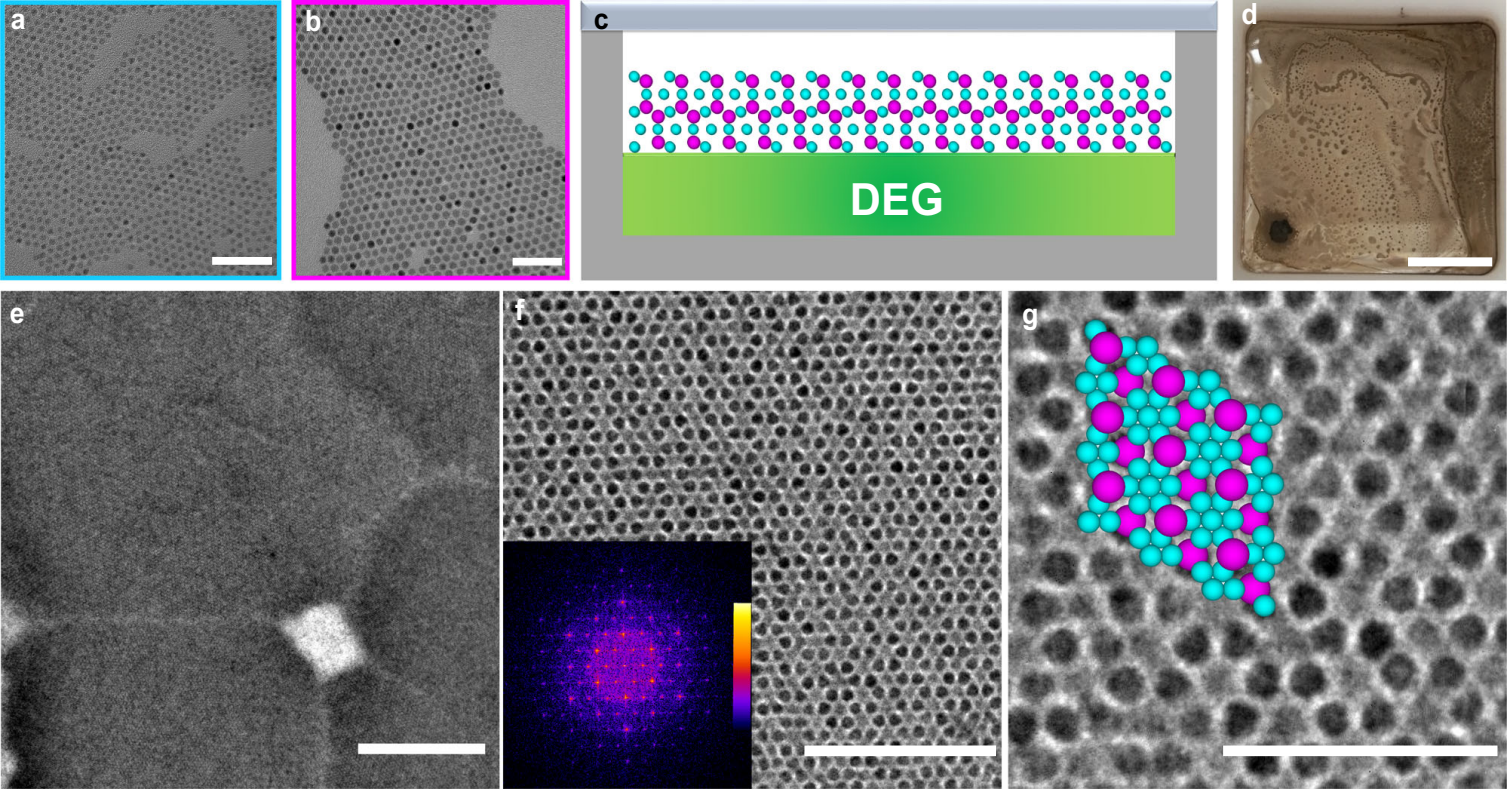
Self-assembled BNSLs in bulk. TEM images of (**a**) *S* CdSe NCs, (**b**) *L* PbSe NCs. (**c**) Schematic illustration and (**d**) an optical image of the BNSLs formation at the liquid–air interface. (**e**) A TEM image of self-assembled BNSL at low magnification. (**f**)–(**g**) BNSL composed of CdSe and PbSe NCs showing an MgZn_2_-like structure viewed along the [0001] projection at different magnifications. The hexagonal symmetry can be visualized from a false-colored Fourier transform (inset of **f**) with a MgZn_2_ structure model (overlay in **g**) viewed along the [0001] projection. Scale bars: 50 nm (**a**), 50 nm (**b**), 0.5 cm (**d**), 500 nm (**e**), 100 nm (**f**) and 50 nm (**g**).

We synthesized monodisperse CdSe NCs (*S*) with a total diameter of 7.7 nm including ligands (5.2 nm core diameter; Fig. 2a) and PbSe NCs (*L*) with a total diameter of 9.9 nm including ligands (7.6 nm core diameter; Fig. 2b), resulting in a size ratio *γ* ≈ 0.78. The van der Waals interaction between two semiconductor NCs (i.e., PbSe and CdSe NCs in our study) in a good solvent is weak and is almost completely screened by the steric repulsion^48^. We, therefore, describe our experimental system with an HS-like potential. To investigate the bulk assembly behavior of the experimental binary components, we let a mixture of the binary NCs assemble on a diethylene glycol (DEG)–air interface^49^ (Fig. 2c), resulting in a solid film floating on the DEG subphase surface (Fig. 2d). The formed BNSL film was then transferred to a TEM grid for further structural analysis (Fig. 2e). We identified the structure of the BNSL following the methodology that is commonly applied in previous studies^6,20,30,48,50–53^. The hexagonal petal-like feature (Fig. 2f, g) and corresponding Fourier transform pattern of a TEM image (inset of Fig. 2f) are in excellent agreement with the [0001] projection of an MgZn_2_ lattice as presented in previous literature^6,20,30,48,50–53^. Moreover, our observation is in line with free-energy calculations in computer simulations showing that the MgZn_2_ phase is the most stable bulk phase for binary HSs^14^. Although the hexagonal pattern is compatible with an MgZn_2_ structure, one cannot exclude possible stacking faults or even the other two Laves structures due to the tiny free-energy difference among the three Laves phases which would induce stacking faults during crystal growth. Recently, the random stacking of Laves phases was also observed in event-driven molecular dynamics simulations of a binary mixture of HSs^54^.

### SA of binary NCs in spherical confinement

**Fig. 3:**
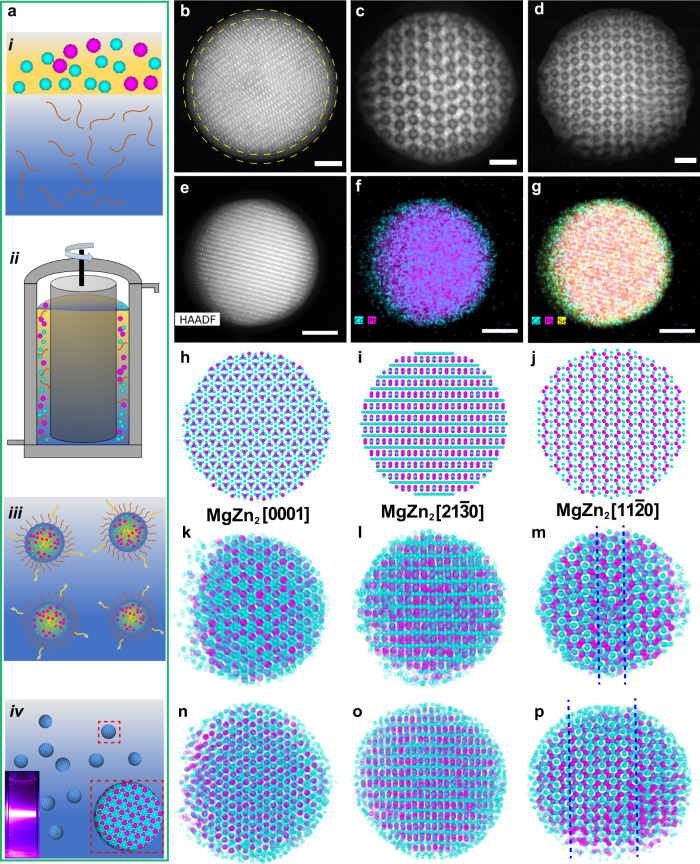
Self-assembled binary Laves SPs. (**a**) Schematic illustration of the formation of Laves phase binary SPs via a drying emulsion droplet method. (**b**) 2D HAADF-STEM image of a self-assembled SP with the core-shell structure highlighted by yellow dashed circles. 2D HAADF-STEM projections of (**c**) a 115 nm-sized SP, (**d**) a 150 nm-sized SP and (**e**) a SP for EDX chemical mapping study projection. Superimposition of (**f**) Pb and Cd and (**g**) of Pb, Cd and Se. Magenta, cyan and yellow represent the distribution of Pb, Cd and Se, respectively. Structure model of a perfect MgZn_2_ SP viewed along the (**h**) [0001], (**i**) [21$\bar{3}$0] and (**j**) [11$\bar{2}$0] projections, respectively. Sparse Sphere Reconstruction (SSR)^39^ renderings of (**k**)–(**m**) a 115 nm-sized SP and (**n**)–(**p**) a 150 nm-sized SP viewed along the (**k**), (**n**) [0001], (**l**), (**o**) [21$\bar{3}$0] and (**m**), (**p**) [11$\bar{2}$0] projections, respectively. Stacking faults in the two SPs are marked with dark blue dashed lines. A digital micrograph of an SP dispersion excited by ultraviolet light (wavelength *λ* = 405 nm) as an inset of (**a**)iv, showing photoluminescence of the SP dispersion at room temperature. Scale bars: 50 nm (**b**), 20 nm (**c**), 20 nm (**d**), 80 nm (**e**)–(**g**). The transparency of the binary species (**k**)–(**p**) was increased for visual clarity. For interactive 3D views of the 115 nm and 150 nm SPs, see Supplementary Data 4–5.

We subsequently let the same binary mixture of PbSe/CdSe NCs self-assemble in slowly drying emulsion droplets (Fig. 3a). We remark that, in contrast to our recent work where a binary mixture with a close to perfect stoichiometry of the Laves phase was used^27^, we used a small excess amount of the *S* CdSe NCs (10% by weight) in our current study. In a typical SA, a cyclohexane dispersion containing 7.7 nm CdSe and 9.9 nm PbSe NCs was mixed with an aqueous solution of surfactants (Fig. 3ai). The mixture was emulsified into an oil-in-water emulsion by a shear force (Fig. 3aii). The resulting emulsion was then evaporated slowly, leading to a slowly increasing packing fraction of the binary NCs in the droplets, which caused crystallization of the binary NCs (Fig. 3aiii–iv). We observed that the self-assembled SPs show photoluminescence when excited by ultraviolet light (Fig. 3aiv inset), which is expected to come from the emission of the CdSe NCs^55^.

We first investigated the self-assembled binary SPs by 2D high-angle annular dark-field scanning transmission electron microscopy (HAADF-STEM) (Supplementary Fig. 1a). Each SP consisted of a highly ordered core composed of both NCs and a few curved outer layers of close-packed CdSe NCs (Fig. 3b, e; Supplementary Fig. 1b). The core-shell structure of the SPs is confirmed by energy-dispersive X-ray spectroscopy (EDX) chemical mapping (Fig. 3e–g; Supplementary Fig. 2). The elemental distributions of lead and cadmium show a core-shell morphology of the SP with respect to the shell of excess CdSe NCs and the core containing both CdSe and PbSe NCs. For SA with an excess of *S* spheres, we investigated two self-assembled SPs with a diameter of 115 and 150 nm, respectively (Fig. 3c–d). By comparing the 2D HAADF-STEM images of the two SPs (Fig. 3c–d) with projections of an MgZn_2_ crystal from three main zone axes (Fig. 3h–j), we found that the stacking is seemingly compatible with that of an MgZn_2_ crystal viewed along the [11$\bar{2}$0] projection (Fig. 3j).

### Quantitative 3D real-space structural analysis

To explore the structure of the self-assembled binary NCs in spherical confinement at the single-NP level, HAADF-STEM tilt series of the two SPs were recorded (Supplementary Movies 1 and 3; Supplementary Figs. 3 and 4). We refer the reader to the Supplementary Information for more details on the reconstructions (Supplementary Figs. 5 and 7). The coordinates of the NCs in the SPs are a direct outcome of the quantitative reconstruction algorithm in which particles with a spherical shape are assumed^39^. Next, the two species were distinguished by calculating the local intensities of all the coordinates in the reconstruction obtained through a Simultaneous Iterative Reconstruction Technique (SIRT)^56^, resulting in two populations based on the atomic number *Z* contrast of the CdSe and PbSe NCs (Supplementary Fig. 6). The crystal structure appeared to match that of the MgZn_2_ crystal when viewed along the [0001] and [21$\bar{3}$0] projections of the coordinates of both SPs (Fig. 3k, l, n, o; Supplementary Figs. 8–10; Supplementary Data 4–5; Supplementary Movies 2 and 4). This was further confirmed by comparing the projections of a spherical model of a perfect MgZn_2_ crystal (Fig. 3h, i; Supplementary Fig. 9). By comparing the [11$\bar{2}$0] projections of both SPs (Fig. 3m, p) with the perfect MgZn_2_ crystal (Fig. 3j), however, stacking faults can be identified.

It is worthwhile to note that in contrast to the binary icosahedral clusters which were self-assembled from the same NC mixture with a number ratio closely matching the perfect stoichiometry of the Laves phases (i.e., $\frac{{N}_{L}}{{N}_{S}}=0.5$)^27^, our core-shell SPs (with an excess of small species) did not show an icosahedral symmetry in any of the SPs observed. Specifically, we found that the number ratio of the core of our SPs matches closely the stoichiometry of the Laves phases (Supplementary Fig. 11). The shell was found to be purely composed of *S* species (i.e., CdSe NCs). The excess CdSe NCs, we infer from our results, most likely alleviated the stress and/or frustration induced by the spherical confinement, resulting in the formation of crystal domains that are similar to binary HSs in bulk systems^13,14^. A recent paper concluded that spherical confinement does not induce icosahedral symmetry in binary SPs^57^. However, based on our experimental observation in this work and our recent study on binary icosahedral clusters^27^, we attribute the absence of icosahedral symmetry in ref. ^57^ to an excess of one of the two species used in the binary crystallization, which forms a shell of colloidal liquid on the outer part of the emulsion droplet and negates the effect of the spherical confinement.

Based on the coordinates of the binary NCs, the radial distribution function (RDF) of the found structures was calculated and compared to the RDFs of the three Laves structures. We performed Monte Carlo (MC) simulations to (locally) equilibrate the three Laves structures of HSs at coexistence pressure, as obtained from the phase diagram for *γ* = 0.76 by Hynninen et al.^14^. The RDFs of both the *L* and *S* species of the reference structures are plotted (Supplementary Fig. 12a, b). In the RDF of the *L* species (Supplementary Fig. 12a) the height ratio of the 4th to 5th peak $\frac{g(r=2.4)}{g(r=2.6)}$ can be used to distinguish the three different Laves structures: for MgZn_2 _$\frac{g(r=2.4)}{g(r=2.6)}\,> \, 1$, for MgNi_2 _$\frac{g(r=2.4)}{g(r=2.6)}\approx 1$ and for MgCu_2 _$\frac{g(r=2.4)}{g(r=2.6)}\,<\,1$. In the RDF of the *S* species (Supplementary Fig. 12b), however, the difference between the distributions is minimal, therefore the RDF of the *S* species cannot be used to distinguish the different Laves crystal structures. Supplementary Fig. 12c shows the RDF of the *L* species in the 115 and 150 nm-sized SPs. Since we find that the ratio of the 4th to 5th peak in the distribution is larger than 1, the RDF is compatible with that of the MgZn_2_ structure. Although the RDF comparison provides structural information, it is limited: the RDF calculations are based on the real-space coordinates of the particles, but is still an ensemble measurement and therefore local structural information remains unrevealed.

**Fig. 4:**
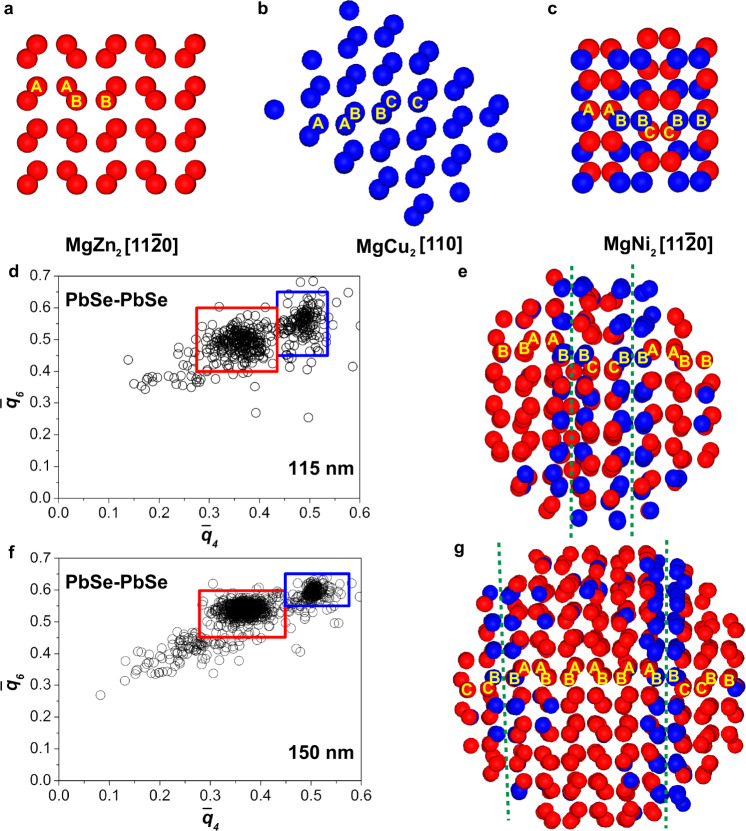
BOP analysis of *L* species. Computer renderings of the *L* species (PbSe NCs) in the Laves phases (**a**) MgZn_2_, (**b**) MgCu_2_ and (**c**) MgNi_2_, where the particles are colored according to their BOP values (see Supplementary Fig. 13). Scatter plots of the BOPs of the *L* species in the (**d**) 115 nm and (**f**) 150 nm-sized SPs, showing two distinct distributions corresponding to the hexagonal diamond (red) and cubic diamond (blue) symmetries. Computer renderings of the *L* species colored according to their BOP values within the red or blue rectangles in (**e**) 115 nm and (**g**) 150 nm-sized SPs, revealing the stacking faults (marked with green dashed lines) in the crystal. Interactive 3D views ((**a**)–(**c**), (**e**) and (**g**)) can be found in Supplementary Data 6–10.

In order to gain more insight into the local structure of the binary SPs, we carried out a BOP analysis to determine the local symmetry of both the PbSe and CdSe NCs. In the BOP analysis, the local symmetry of every particle with respect to its neighbors is determined through the calculation of its spherical harmonics^58^. In this work, we used the averaged local BOPs ${\bar{q}}_{4}$ and ${\bar{q}}_{6}$, which are sensitive to local cubic and icosahedral symmetry, respectively^58^, for which we also take into account the second shell of neighbors around each particle^40^. In order to get an estimate of the upper limit of the effect of fluctuations in the particle positions with respect to their ideal lattice sites on the BOP analysis, we use the equilibrated Laves crystal structures. The effect of the fluctuations on the BOP analysis is most likely less than the effects of polydispersity in our system since our SPs were compressed to the highest density. Next, we distinguished the three Laves phases by assigning the local symmetry of each particle according to specific areas in the ${\bar{q}}_{6}-{\bar{q}}_{4}$ scatter plot of the *L* species (Supplementary Fig. 13). The different symmetries of the *L* species in the MgZn_2_ phase (hexagonal diamond) and MgCu_2_ phase (cubic diamond) lead to a distinct grouping of these two Laves phases in the scatter plot (Supplementary Fig. 13a). The BOP values of the *L* species in MgNi_2_ are split between the above-mentioned two symmetries as this Laves phase can be considered as a mixture of the other two Laves phases. Figure 4a–c shows the *L* species in the three Laves phases with assigned symmetries: the *L* species in MgZn_2_ and MgCu_2_ have the same symmetries throughout the whole structure, whereas in the MgNi_2_ structure the hexagonal diamond (red) and cubic diamond (blue) symmetries of the *L* species alternate (Supplementary Data 6–8). Figure 4d, f shows the scatter plots calculated from the coordinates of the *L* species obtained through the electron tomographic reconstruction of both the 115 nm and 150 nm-sized SPs. When assigning the symmetry of each *L* particle according to their BOP values, we find that most *L* species exhibit a hexagonal diamond symmetry, with stacking faults with cubic diamond symmetry present in both SPs (Fig. 4e and g; Supplementary Data 9–10).

**Fig. 5:**
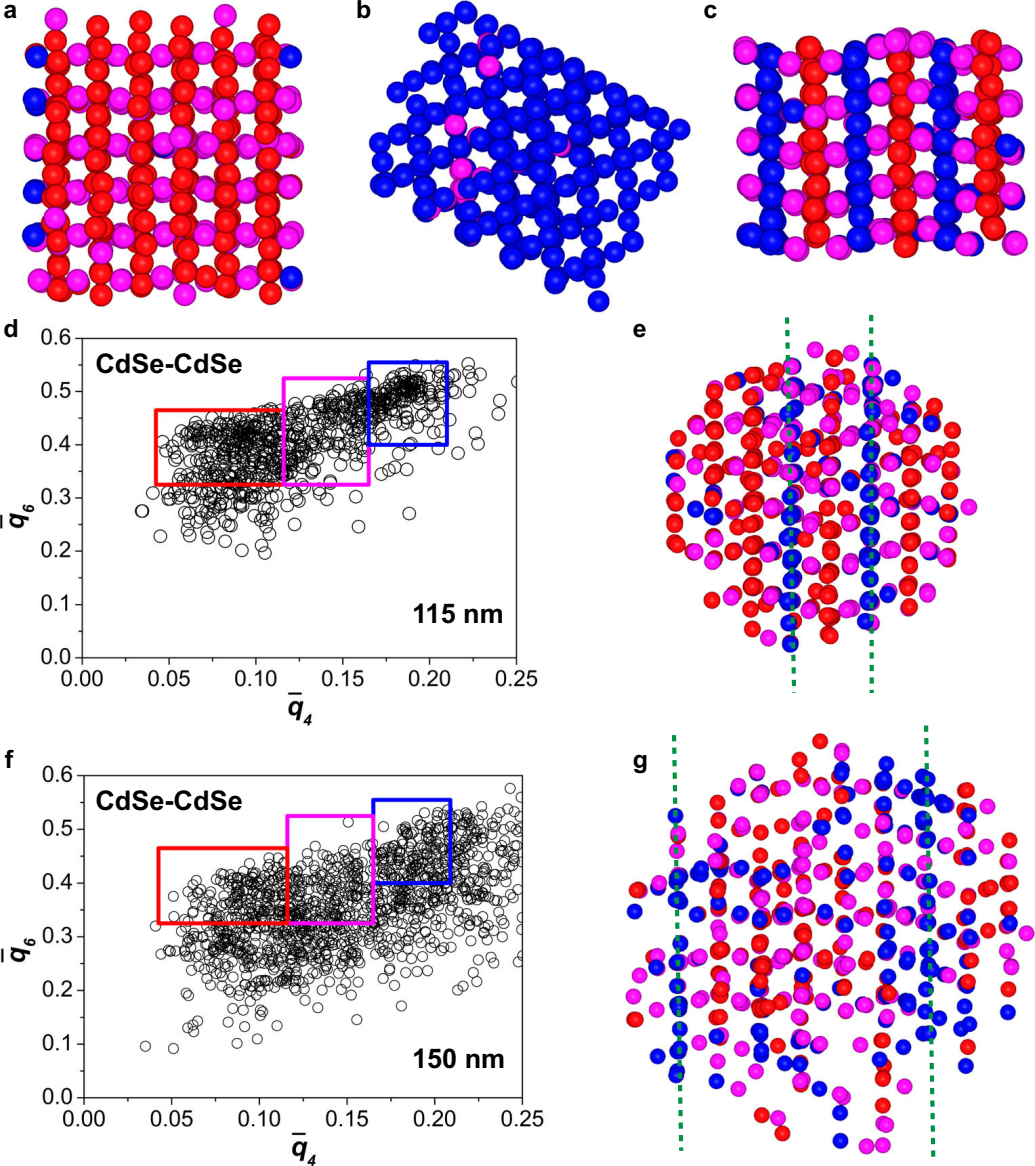
BOP analysis of *S* species. Computer renderings of the *S* species (CdSe NCs) in the Laves phases (**a**) MgZn_2_, (**b**) MgCu_2_ and (**c**) MgNi_2_, where the particles are colored according to their BOP values (Supplementary Fig. 14). Scatter plots of the BOPs of the *S* species in (**d**) 115 nm and **f** 150 nm-sized SPs, showing less well-defined distributions compared with the *L* species (Fig. 4). Computer renderings of the *S* species with BOP values within the blue, magenta or red rectangles in (**e**) 115 nm and (**g**) 150 nm-sized SPs, revealing the stacking faults (marked with green dashed lines) in the crystal. Interactive 3D views (**a**–**c**, **e** and **g**) can be found in data Supplementary Data 11–15.

For the *S* species in the reference Laves structures, a more complex scatter plot is obtained (Supplementary Fig. 14). Assigning the local symmetry to each *S* particle reveals a clear difference between the three Laves structures: all the *S* species have the same symmetry in the MgCu_2_ phase, whereas the *S* species have two and three different symmetries in the MgZn_2_ and MgNi_2_ phases, respectively (Fig. 5a–c; Supplementary Data 11–13). The BOP values in the ${\bar{q}}_{6}-{\bar{q}}_{4}$ scatter plots of the *S* particles in the two SPs are less grouped in distinct distributions compared to those of the *L* ones (Fig. 5d, f). However, assigning the local symmetries to the *S* species, reveals the stacking faults (Fig. 5e, g; Supplementary Data 14–15) similar to the ones found from the *L* species BOP analysis in both SPs (Fig. 4e, g). Interestingly, the stacking of both the *S* and *L* species remains identical throughout the structure, making the stacking faults continuous transitions from one structure to the other for both species.

As mentioned above, the BOP values in the ${\bar{q}}_{6}-{\bar{q}}_{4}$ scatter plots of the *S* species in the two SPs are significantly less grouped in distinct clouds compared with those of the *L* species, resulting in less clearly defined planes of similar colored particles (Fig. 5d–g). The size ratio *γ* = 0.78 of our system is on the lower end of the size ratio range where the Laves phases form. This means that the *S* particles have more room to move relative to the *L* particles, leading to less well-defined coordinates of the *S* particles with respect to the *L* particles and therefore a less well-defined distribution of the BOP values as compared with an ideal Laves lattice. Alternatively, the less well-defined distribution of the BOP values of the *S* species could arise from the smaller *Z* contrast of the species, which might increase the positional error in the reconstruction of those species, resulting in a decrease in order. In addition, the distribution of the BOP values of the *S* particles in the 150 nm-sized SP is less concentrated than the values of the 115 nm-sized SP (Fig. 5d, f). We speculate this is due to the large size of the 150 nm-sized SP. When the SP is thick, it becomes more challenging to retrieve the positions of all particles with lower atomic number *Z* contrast (i.e., *S* particles (CdSe NCs) in this case) from the tomographic reconstruction, possibly resulting in missing particles, which leads to lower BOP values of the identified *S* spheres. This is supported by calculating the number ratio of *L* to *S* species ($\frac{{N}_{L}}{{N}_{S}}=0.5$ for Laves phases) inside the binary crystalline cores for the 115 nm and 150 nm-sized SPs: 0.50 and 0.57, respectively, indicating missing *S* species in the 150 nm-sized SP. The number ratio of the *L* and *S* spheres inside the binary crystal region of the 115 nm-sized SP matches the ratio of the perfect Laves crystal ($\frac{{N}_{L}}{{N}_{S}}=0.5$; Supplementary Fig. 11): this observation is in contrast with earlier work on HS binary NaCl crystals^59,60^. In previous studies, we demonstrated that for binary HSs with a size ratio *γ* ≈ 0.3, forming NaCl-type binary crystals, the *S* species do not fill their stoichiometric number of lattice positions, unless their osmotic pressure is quite high. The crystals found can therefore be better described as an ISS with tunable lattice doping^59,60^. This discrepancy of the stoichiometry in the binary structure was due to the increased entropy associated with this ‘imperfect’ lattice filling. Future work is needed to reveal why this behavior is so different for these two binary crystals. Moreover, a recent simulation study showed that the number ratio of a bulk MgZn_2_ phase of HSs with a size ratio of 0.82 is <0.5 due to an equilibrium concentration of anti-site defects^61^, which was not observed in the present study.

The BOP analysis identified the stacking faults in both SPs to have local MgCu_2_ symmetry, for both the *L* and *S* species (Fig. 4e, g; Fig. 5e, g). The stacking faults consisted of a pair of *L* species from two adjacent triple-line (*t* or *t’*) stacks (e.g., *BB* in Fig. 4e, g) and a single line of *S* species (particles align along the blue dashed lines in Fig. 5e, g), all with MgCu_2_ symmetry. This is equivalent to an insertion of a *s t* unit in an MgZn_2_ structure (e.g., *s t s t’ s t ****s t*** *s t’ s t s t’* instead of *s t s t’ s t s t’ s t s t’*). In the 150 nm-sized SP the stacking faults are five *L* pairs apart, leading to two MgZn_2_ zones which are slightly shifted with respect to each other (i.e., ...*AABBAA*... and ...*BBCCBB*..., see Fig. 4g). In the 115 nm-sized SP, however, the two defect lines are only one pair of *L* spheres apart. This can therefore be seen as a single mis-stacking of the *CC* layer in an otherwise perfect MgZn_2_...*AABB*... stacking (see Fig. 4e). Since the stacking faults are only one pair of *L* spheres away from each other, one can also interpret the majority of the 115 nm-sized SP to be the MgNi_2_ structure. This emphasizes the importance of thick enough samples for the proper distinction of the MgZn_2_ and MgNi_2_ structures. The fact that we do not observe any MgCu_2_ domains (...*AABBCC*...) might be due to the limited number of stacked layers studied here. This suggests that superlattices identified as pure MgZn_2_ in earlier studies might very well have been a mixture of the three Laves phases, similar to the random stacking of hexagonally closed-packed layers in single-species HSs^16^. Further study on the stacking probability, as has been done for single-species HSs^16^, requires the real-space analysis of larger crystals to obtain more stacking layers. We foresee that the morphology of SPs enables the analysis of stacking probability of binary colloidal crystals in general.

## Discussion

We further compare our observations on the colloidal Laves phase crystals of hard particles to that of their atomic counterparts. Stacking faults have also been found in intermetallic alloys upon synchroshear or upon aging^11,12^. In addition, it has been found that an excess of one of the binary species can induce the formation of other planar defects such as coherent planar *μ* phases (or structural motifs of the *μ* phase) or Laves-related phases such as *LS*_3_ and *L*_4_*S*_7_ with excess atoms originating from off-stoichiometry^11^, but these were not found in our current study. For single-component crystals, the free energy of a stacking fault in close-packed HS colloidal systems is four orders of magnitude smaller than in FCC atomic systems^17^. We expect the stacking fault free-energy difference between atomic and HS colloidal Laves phases to be of similar order and therefore a higher abundance of these stacking faults in the HS colloidal Laves phases. Still, it would be intriguing to probe in future experiments on our binary SPs if further off-stoichiometry changes the probability and/or types of planar defects.

It has been shown that in MgZn_2_ crystals, the formation of dislocations with the MgCu_2_ symmetry can be described as the simultaneous shear of two sub-layers along different directions, a mechanism known as synchroshear^11,62,63^. The synchroshear can result in dislocations with MgCu_2_ symmetry with varying thickness: ... *s t’ s t s t s t s t’*... (also referred to as an *I*_2_ stacking fault^64^) and ... *s t’ s t s t s t s t s t s t’*..., so with either three or five repeating *s t* units^62^. Further synchroshear applied to the *I*_2_ stacking fault can form the twin-like stacking fault *T*_2_, resembling the MgNi_2_ structure^64^, corresponding to the structure found in our experimental 115 nm-sized SP. In the 150 nm-sized SP, we have only found dislocations with a thickness of two repeating *s t* units, which is the simplest stacking fault possible in the Laves phases and cannot be formed from a perfect MgZn_2_ crystal using the synchroshear mechanism.

So far, only a few simulation studies to date have reported crystallization of Laves phases in a binary HS mixture, either from an unstable fluid phase so that the phase transformation proceeds via a spinodal-like crystallization^54^ or by using seeding simulations^65^. Observation of Laves phase nucleation is extremely rare as it is severely inhibited by glassy dynamics^65^. Moreover, frustrating the initial conditions by using spherical confinement or off-stoichiometry conditions will make the observation of Laves phase nucleation even more difficult. Our recent work showed that the formation of binary icosahedral clusters is driven by the structured layering of varying species in distinct concentric shells^27^. We speculate that the stoichiometry of the fluid will change the layering of the fluid and therefore plays a crucial role on the nucleation and growth mechanism, resulting into different SP structures. In addition, we believe that the stacking faults in the MgZn_2_ crystal structure formed in the SPs as a result of mis-stacking during crystal growth, which have not been able to anneal out within the time-scale of the emulsion droplet drying, a process which was found to be slow for single-species crystals of HS particles^15^.

To conclude, we quantitatively studied the 3D real-space structures of two SPs with bulk-like crystal structures formed by the SA of a binary mixture of CdSe/PbSe NCs with a size ratio of 0.78 in spherical confinement. The formation of icosahedral symmetries was suppressed by using an excess of small species (CdSe NCs), where the excess particles formed a shell around a bulk-like crystalline core. Our approach enabled us to obtain real-space coordinates of a bulk-like binary crystal composed of over 50 layer at the single-nanoparticle level by advanced electron tomography, which was hardly achieved till now. We think it is likely that our approach can also be applied to other binary crystal systems. With the coordinates of both species in the crystal obtained it was possible to analyze the stacking order in the SPs using a bond-order parameter analysis. We highlight that the structure found should be characterized as a mixture of the hexagonal Laves phases as a result of small free-energy differences between the Laves phases, similarly as single HSs are prone to form randomly stacked close-packed layers. We showed that the stoichiometry in the binary Laves phase core of our SPs was close to that of a perfect Laves phase (i.e. 0.5), in contrast to earlier work on binary hard-sphere NaCl crystals with a size ratio of 0.3, where an ISS structure with a composition different from that of a perfect NaCl structure was found^59,60^.

Connecting with our recent work on binary icosahedral clusters^27^, we remark that the interplay between spherical confinement, number ratio and size ratio of the binary components allows us to have full control over the structure (i.e., stacking and symmetry) of the SPs. The great tunability of the structure makes it possible to finely tailor the properties of the SPs. In addition, the HS potential of the binary building blocks enables the preservation of the structure of the SPs after ligands removal or ligands exchange to smaller ligands, making it possible to realize strongly coupled electronic properties (e.g., conductivity) of the binary SPs, as has been demonstrated for BNSLs^50,66^. This opens up a new avenue to build up quantum dots-based devices with SPs in future work. We also expect to extend our SA study in drying emulsion droplets to grow Laves SPs using binary colloidal mixtures of micron-sized HSs (e.g., silica-titania colloids), as the HS interactions are scale and system independent. The micron-sized HSs can be visualized by light micro- and nanoscopy in real space, thus shedding more light on the formation of defects. Our methodology will be applicable to a broad range of self-assemblies, which will provide new insights into many more structure-property relationships.

## Methods

### NC syntheses

We synthesized 5.2 nm CdSe NCs (7.7 nm total diameter; a total polydispersity of 2%) and 7.6 nm PbSe NCs (9.9 nm total diameter; a total polydispersity of 1%) according to reported protocols^67,68^ with minor modifications. The NC syntheses were performed in a nitrogen atmosphere using standard Schlenk line techniques. Prior to SA, the NCs were precipitated with isopropanol, isolated by centrifugation and redispersed in *n*-hexane. The amount of free ligands can be considered to be negligible after proper purification such that they did not play a substantial role during the SA. Details of the NC syntheses can be found in Supplementary Materials and Methods Section 1 and 2.

### SA of binary NCs in bulk

BNSLs were prepared at a liquid–air interface^49^ in a glovebox that maintained oxygen- and moisture-free conditions. In all, 5.2 nm CdSe and 7.6 nm PbSe NCs were separately dispersed in *n*-hexane at a concentration of 10 mg/mL. The two samples were mixed with a volume ratio of 0.9, which corresponds to a number ratio as found in the perfect Laves phases. Next, 10 μL of the mixture was drop-cast onto the surface of DEG in a square Teflon well (1.5 cm × 1.5 cm × 1 cm). A glass slide was placed to cover the well and reduce the evaporation rate of hexane. After 30 minutes, a solid film was formed on the liquid–air interface and was then transferred onto a carbon-coated Cu TEM grid. The grid was then dried under vacuum at room temperature (RT) to remove residual DEG.

### SA of binary NCs in spherical confinement

For a typical Laves SP with a core-shell structure, 6.5 mg of PbSe NCs and 6.5 mg of CdSe NCs, corresponding to an excess amount of CdSe NCs with respect to the perfect Laves phases, were redispersed in 1.0 mL of cyclohexane and added to a mixture of 400 mg of dextran and 70  of sodium dodecyl sulfate in 10 mL of de-ionized (DI) H_2_O. The resulting emulsion was agitated by shear with a shear rate of 1.56 × 10^5^ s^−1^, using a Couette rotor-stator device (gap spacing 0.100 mm) following the procedure and home-built equipment described by Mason and Bibette^69^. The emulsion was then evaporated at RT, whereas sedimentation was prevented by mixing the emulsion using a VWR VV3 vortex mixer for 48 h. The resulting SPs suspension was purified by centrifugation with a Relative Centrifugal Force of 489 × *g* for 15 mins using an Eppendorf 5415C centrifuge, followed by re-dispersing in DI H_2_O. The aforementioned procedure was repeated twice.

### Electron microscopy (EM) sample preparation and 2D EM measurements

To prepare a sample for conventional 2D EM imaging and electron tomography analysis, 3 μL of the SPs suspension in DI H_2_O was deposited on a Quantifoil (2/2, 200 mesh) copper grid and plunge-frozen in liquid ethane using a Vitrobot Mark2 plunge freezer at temperatures ~90 K. Prior to EM analysis, the sample was then freeze-dried over a period of 8 h under vacuum at 177 K and subsequently allowed to warm to RT.

Conventional TEM measurements were conducted on a JEOL 1400 microscope operated at 120 kV and an FEI Talos F200X TEM equipped with a high-brightness field emission gun (X-FEG) operated at 200 kV. 2D HAADF-STEM measurements were performed on an FEI Talos F200X TEM operated at 200 kV.

2D EDX chemical mapping measurements were performed using an FEI Talos F200X TEM, equipped with X-FEG and a Super-X G2 EDX detector operated at 200 kV. Images and elemental EDX maps were acquired using Bruker Esprit analytical and imaging software in scanning transmission mode. Elemental EDX maps of 400 × 400 pixels, and 408 × 349 pixels were acquired with a 15-min acquisition time to achieve a good signal-to-noise ratio.

### Electron tomography

Electron tomography measurements were performed using a ‘cubed’ Thermo Fisher Scientific Titan TEM, operated at 300 kV in HAADF-STEM mode with a semi-convergence angle of 8 mrad, corresponding to a probe size of 0.15 nm and a depth of focus of 110 nm. A Fischione model 2020 single tilt holder was used for the acquisition of the tilt series. Before tilt series acquisition, we stabilized SPs by pre-illuminating them at different tilting angles at an low magnification with a beam current of ~40 pA. Such initial tilt series were obtained with an acquisition time of 2 μs and an increment of 2^∘^. This is similar to the so-called “beam-showerʼ or “pre-exposureʼ^35,70^, in order to prevent significant sample shrinkage during the tilt series acquisition. By doing so, we note that the SPs can be stabilized in a homogeneous manner. A tilt range from −72^∘^ to +66^∘^ for the 115 nm SP and −76^∘^ to +62^∘^ for the 150 nm SP, with an increment of 2^∘^, respectively, were applied. A beam current of ~100 pA was used during the tilt series acquisition. To minimize sample drift and distortion during image acquisition, six frames and 10 frames at each titling angle were recorded with a 2 μs dwell time for the 115 nm and 150 nm SPs, respectively, which were aligned using an iterative registration algorithm followed by computing the mean. The registration algorithms started by aligning all projections to the first one acquired, using a subpixel cross-correlation algorithm^71^. After the first iteration, a mean projection was computed from the aligned projections and was then used as a reference image for the following iterations. In each following iteration, all projections were aligned to the mean projection computed at the end of the previous iteration. The procedure was repeated until convergence was reached and no more cumulative shifts were detected. The final projection with a high signal-to-noise ratio without deformation induced by drifting was obtained. The total dose of the tilt series for the 115 nm and 150 nm SP was 4.2 × 10^7 ^e^−^ nm^−2^ and 2.6 × 10^7 ^e^−^ nm^−2^, respectively.

### Tomographic reconstruction

The tilt series were aligned using cross-correlation routines^71^ implemented in Matlab. The SIRT^56^ algorithm, which is implemented in ASTRA toolbox^72,73^, was applied to obtain a preliminary reconstruction of the tilt series yielding morphological information about the 3D morphology of the SPs. However, in the presence of close-packed particles, conventional SIRT is not sufficient to achieve the reconstruction resolution necessary to segment individual particles and obtain structural information. In order to get coordinates of the binary species from the SPs, we applied the sparse sphere reconstruction (SSR) algorithm^39^, which has been developed for the quantitative reconstruction of assemblies of spherical particles, to two tilt series of SPs.

The two types of particles in the SPs differ only slightly in size and density. At the resolutions used for the reconstructions, the difference in size between the two species was less than two pixels. Therefore, in practice, the binary species are equivalent to the reconstruction algorithm. The SSR algorithm could be applied using the approximation of monodisperse particles and only one size (estimated from the SIRT reconstruction, as the size of the *S* species) was then used as prior knowledge. Extracting the local maxima positions produces a list of coordinates of both species indistinctively.

After the coordinates of all particles were determined by the SSR technique, the particles were assigned to be a *S* (CdSe NC) or *L* (PbSe NC) species. To distinguish the binary species, a spherical neighborhood around each center coordinate and with a radius equivalent to one of the *S* species (CdSe NCs) was extracted from the SIRT volume. For each point, an average gray value was calculated for these neighborhoods. Since the tilt series were acquired in HAADF-STEM mode, the different atomic numbers of the cations create a different contrast in the SIRT volumes, which yielded two different intensity distributions. The intensity distributions were fitted with two Gaussian distributions, and the intersection of the two curves was taken as the threshold value to distinguish the two species (Supplementary Fig. 6). For both the 115 nm and 150 nm SPs, the overlap of the two curves gives an estimate of the percentage of particles that might be misidentified (~1%). We compared the computed forward projections of the 115 nm SP by the SIRT (Supplementary Movie 5) and the SSR (Supplementary Movie 6), which show a great agreement with each other (Supplementary Fig. 7). Details of the SSR reconstructions can be found in Supplementary Materials and Methods Section 3.

### Radial distribution function

The RDF *g*(*r*) of the NCs in spherical confinement were calculated as follows: first, the distances between all pairs of particles were calculated, next the volume of the droplet was estimated by wrapping the particles with a convex hull. This hull was then randomly filled with *N*_*ig*_ ideal gas particles, whose pair distances were used to divide the distribution of distances of the *N*_*np*_ NCs. In case *N*_*ig*_ ≠ *N*_*np*_, the distribution was normalized by a factor of ${({N}_{ig}/{N}_{np})}^{2}$. For the *g*(*r*) of the Laves phases from computer simulations, the ideal gas particles were generated in a box (instead of a convex hull) defined by the minimum and maximum values of the coordinates of the data sets.

### BOP analysis

Averaged local BOPs ${\bar{q}}_{4}$ and ${\bar{q}}_{6}$ were used to determine the symmetry of the individual NCs, for the *L* (PbSe NCs, Supplementary Fig. 13) and *S* (CdSe NCs, Supplementary Fig. 14) species separately^40^. First, a set of numbers was calculated for every particle, based on spherical harmonics *Y*_*lm*_:

1 $${q}_{lm}(i)=\frac{1}{{n}_{c}(i)}\mathop{\sum }\limits_{j=1}^{{n}_{c}(i)}{Y}_{lm}({{\bf{r}}}_{ij}),$$

where *n*_*c*_(*i*) is the number of nearest neighbors of particle *i*, *l* an integer (in our case 4 or 6), *m* an integer running from −*l* to *l* and **r**_*ij*_ the unit vector pointing from particle *i* to particle *j*. The nearest neighbors were defined as the particles within cut-off distance *r*_*c*_ from particle *i*. This cutoff was determined from the first minimum of the RDF *g*(*r*) (Supplementary Fig. 12) of the species in the BOP analysis, corresponding to *r*_*c*_ ≈ 1.4*d*. This minimum was estimated by eye and small variations in its value did not have a significant effect on the bond-order analysis. Next, the *q*_*lm*_ set of numbers of particle *i* was averaged with the values of its neighbors:

2 $${\bar{q}}_{lm}(i)=\frac{1}{{n}_{c}(i)+1}\mathop{\sum }\limits_{k=0}^{{n}_{c}(i)+1}{q}_{lm}(k),$$

where *k* = 0 denotes the particle *i* itself. This set of numbers then yields the averaged local BOP:

3 $${\bar{q}}_{l}(i)=\sqrt{\frac{4\pi }{2l+1}\mathop{\sum }\limits_{m=-l}^{l}{\left|{\bar{q}}_{lm}(i)\right|}^{2}}.$$

In the BOP calculations, particles too close to the surface of the SP were excluded from the calculation of ${\bar{q}}_{l}$ since their lower amount of nearest neighbors influences the parameter values. In practice, this corresponds to two layers of particles excluded from the surface. In order to identify the crystal structure of the SPs we compared the ${\bar{q}}_{6}-{\bar{q}}_{4}$ scatter plot of the simulated Laves phases at coexistence density, of both the *L* *and* *S* species with the experimental data sets. By coloring the particles in the computer reconstructions according to their position in the ${\bar{q}}_{6}-{\bar{q}}_{4}$ scatter plot and therefore their local symmetry, the local crystal structure could be identified. For the *L* particles two regions were specified: $0.4\,<\,{\bar{q}}_{6}\,<\,0.6$ & $0.285\,<\,{\bar{q}}_{4}\,<\,0.45$ (red, hexagonal diamond symmetry) and $0.445\,<\,{\bar{q}}_{6}\,<\,0.645$ & $0.45\,<\,{\bar{q}}_{4}\,<\,0.57$ (blue, cubic diamond symmetry). For the *S* particles three regions were defined: $0.4\,<\,{\bar{q}}_{6}\,<\,0.555$ & $0.165\,<\,{\bar{q}}_{4}\,<\,0.21$ (blue), $0.325\,<\,{\bar{q}}_{6}\,<\,0.525$ & $0.116\,<\,{\bar{q}}_{4}\,<\,0.165$ (magenta) and $0.325\,<\,{\bar{q}}_{6}\,<\,0.465$ & $0.0425\,<\,{\bar{q}}_{4}\,<\,0.116$ (red).

### MC computer simulations

We performed MC computer simulations in the isothermal-isobaric (NPT) ensemble on the three perfect Laves crystal structures for a binary HS mixture with size ratio *γ* = 0.76. The simulations were performed at a pressure close to the melting pressure, $\beta P{\sigma }_{L}^{3}=26.0$, of the Laves phase as obtained from the phase diagram of binary HSs for size ratio *γ* = 0.76^14^. This was done in order to obtain a distribution in the values of the BOPs ${\bar{q}}_{4}$ and ${\bar{q}}_{6}$ that should be broad enough to distinguish the Laves-ordered NCs from a high density disordered fluid as far as possible. The NPT simulations were equilibrated for 50,000 cycles and anisotropic volume change moves were used for the hexagonal Laves crystal structures, i.e., the MgZn_2_ and MgNi_2_ structures.

## Supplementary information

Supplementary Information

Description of Additional Supplementary Files

Supplementary Data 1

Supplementary Data 2

Supplementary Data 3

Supplementary Data 4

Supplementary Data 5

Supplementary Data 6

Supplementary Data 7

Supplementary Data 8

Supplementary Data 9

Supplementary Data 10

Supplementary Data 11

Supplementary Data 12

Supplementary Data 13

Supplementary Data 14

Supplementary Data 15

Supplementary Movie 1

Supplementary Movie 2

Supplementary Movie 3

Supplementary Movie 4

Supplementary Movie 5

Supplementary Movie 6

## Data Availability

The data of this study are available from the corresponding authors upon reasonable request. Source data are provided with this paper.

## Source data

Source Data

## Code availability

The tomographic reconstruction, RDF and bond-order parameter codes used in this study are available upon reasonable request.
