# Supplementary material for: Quantitative 3D real-space analysis of Laves phase supraparticles
Source: Nat Commun. 2021 Jun 25;12:3980. doi: 10.1038/s41467-021-24227-0 (PMC8233429; doi:10.1038/s41467-021-24227-0)
Supplement: Supplementary file 1 — Supplementary Information [file 41467_2021_24227_MOESM1_ESM.pdf]

Quantitative 3D real-space analysis of Laves phase supraparticles

Da Wang[1,‖,†,*], Ernest B. van der Wee[1,‖,‡], Daniele Zanaga[2,#], Thomas Altantzis[2], Yaoting Wu[3], Tonnishtha Dasgupta[1], Marjolein Dijkstra[1], Christopher B. Murray[3,4], Sara Bals[2,5], and Alfons van Blaaderen[1,*]

[1]*Soft Condensed Matter, Debye Institute for Nanomaterials Science, Utrecht University, Princetonplein 5, 3584 CC, Utrecht, The Netherlands.*

[2]*Electron Microscopy for Materials Science (EMAT), University of Antwerp, Groenenborgerlaan 171, 2020 Antwerp, Belgium.*

[3]*Department of Chemistry, University of Pennsylvania, Philadelphia, PA 19104, United States.*

[4]*Department of Materials Science and Engineering, University of Pennsylvania, Philadelphia, PA 19104, United States.*

[5]*NANOlab Center of Excellence, University of Antwerp, Belgium.*

[‖]*These authors contributed equally to this work.*

[†]*Present address: Electron Microscopy for Materials Science (EMAT), University of Antwerp, Groenenborgerlaan 171, 2020 Antwerp, Belgium.*

[‡]*Present address: Department of Imaging Physics, Faculty of Applied Sciences, Delft University of Technology, Lorentzweg 1, 2628 CJ, Delft, The Netherlands.*

[#]*Present address: Vlaamse Instelling voor Technologisch Onderzoek (VITO), Belgium.*

[*]*e-mail: dawangcolloid@gmail.com; a.vanblaaderen@uu.nl*

Supplementary Methods

Section 1 Chemicals

Chemicals used were: dextran from Leuconostoc mesenteroides (Sigma Aldrich, mol. wt. 1,500,000-2,800,000), cyclohexane (Sigma Aldrich, ≥99.8%), *n*-hexane (Sigma Aldrich, ≥99.5%), 1-octadecene (1-ODE, Sigma Aldrich, 90%) Sodium dodecyl sulfate (SDS, Sigma Aldrich, ≥99.0%), oleic acid (OA, Sigma Aldrich, ≥99.0%), isopropanol (Sigma Aldrich, ≥99.5%), diphenylphosphine (Sigma Aldrich, >90%), trioctylphosphine (TOP, Sigma Aldrich, 97%), selenium shot (Se, Alfa Aesar, 99.999%), lead (II) oxide (PbO, Sigma Aldrich, 99.999%), cadmium oxide (CdO, Sigma Aldrich, ≥99.99%), toluene (Sigma Aldrich, anhydrous, 99.8%), diethylene glycol (DEG, Sigma Aldrich, 99%). For de-ionized water (DI H_2O) a Millipore Direct-Q UV3 reverse osmosis filter apparatus was used (18 MΩ at 25 °C).

Section 2 Nanocrystal (NC) syntheses and self-assembled supraparticles (SPs)

Section 2.1 CdSe NCs

5.2 nm CdSe NCs were synthesised by cation exchange from 5.2 nm PbSe template based on reported recipes[1]. 750 mg of CdO, 5.0 mL of OA and 25 mL 1-ODE were mixed and degassed at 105 °C for 30 minutes. Then the mixture was heated to 250 °C in a nitrogen atmosphere to get a clear solution. The solution was kept at 250 °C for 20 minutes, then allowed to cool down to 105 °C. It was degassed again for 1 hour and then heated to 210 °C. 150 mg of PbSe NCs were dispersed in 3.0 mL of toluene and swiftly injected into the reaction flask. The solution turns red in a few seconds, indicating the formation of CdSe NCs. The reaction was kept at 210 °C for 45 minutes and then cooled down to room temperature. The as-synthesised CdSe NCs were purified with isopropanol three times, dispersed in *n*-hexane and stored in a glove box.

Section 2.2 PbSe NCs

7.6 nm PbSe NCs were synthesised following reported recipes[2]. Specifically, 0.895 g of PbO, 3.0 mL of OA, and 20 mL of 1-ODE were mixed and degassed at 105 °C for 1.5 hours. Then the solution was heated to 105 °C in a nitrogen atmosphere. 71 µL of diphenyl phosphine was mixed with 8.0 mL of trioctylphosphine-selenium (TOP-Se) solution (1 M). The mixture was swiftly injected into the reaction flask. The NCs were allowed to grow at 105 °C for 10 minutes and then quenched by an ice bath. The as-synthesised PbSe NCs were purified with isopropanol three times, dispersed in *n*-hexane and stored in a glove box.

Section 3 Details of the SSR reconstruction

Classical reconstruction algorithms such as Weighted Back-projection (WBP) and Simultaneous Iterative Reconstruction Technique (SIRT) yield 3D images that suffers from missing wedge[5]. Advanced algorithms for instance Total variation minimization (TVM)[3] or Discrete algebraic reconstruction technique (DART)[4] have recently been proposed to improve the reconstruction results and to compensate for missing wedge artefacts through the implementation of prior knowledge. However, the above-mentioned algorithms are still limited for the 3D investigation of complex assemblies, because the existing algorithms do not yield sufficient resolution to allow one to study the structure on a single-particle level by application of a threshold. It becomes even more challenging for assemblies with a high number of particles where the particles are in contact with each other. A manual segmentation step would be quite extremely time consuming. In addition, manual segmentation is a subjective process which may fail to interpret the structure in a quantitative manner. Therefore, we applied the SSR algorithm which tackles the challenges of reconstructions of large-sized assemblies[5–7]. Moreover, we have demonstrated in our previous work that the SSR algorithm recovers the lost information induced by the missing wedge better than the SIRT does. This was better observed for the smaller assemblies, for which projections were obtained over small tilt ranges causing more severe missing wedge artefacts[5].

We invite the readers to refer to our paper[5] in which we described full details of the SSR algorithm and showed several study cases in an intuitive manner.

Basically, the SSR technique works as a two-step reconstruction. The first step solves a mathematical reformulation of the conventional reconstruction problem, where we introduced prior knowledge of spherical particles. This step can be considered as a simultaneous reconstruction/deconvolution by spherical kernel, producing a sparse solution consisting of centers of spheres. Due to experimental limitation induced by noise and discrete tilt angle acquisitions *etc.*, in contrast to a sparse one, the algorithm yields a continuous solution with three-dimensional gradients of intensity around the estimated particles position. In a second step, these local maxima of the intensity in the obtained sparse solution are extracted, determining the position of the particles. Afterwards, the species identification (*i.e.* chemical compositions) is realised by extracting the histogram by the SIRT reconstruction. Finally, the positions of the particles are convolved by a sphere of the gray values and sizes as determined by the SIRT reconstruction.

In the current study, the binary species are of similar size but of different contrast. The SSR reconstruction works in the same way for both species. We obtained blobs of 3D probability where the central maxima was the position of the particles, being independent of its chemical composition. The different atomic number of the cations create a different contrast in the volume of SIRT reconstructions. For each particle position we extracted a neighbourhood of the SIRT reconstruction and measured the average intensity. The obtained intensity distributions were fitted were fitted with two Gaussian distributions as shown in the Supplementary Fig. 6. We used the intersection of the two curves as the threshold value to distinguish the two species. The positions of the binary species were then convolved by a sphere of the gray values and sizes as determined by the SIRT reconstruction.

We computed the forward projections of the 115 nm supraparticle by the SIRT (Supplementary Movie 5) and the SSR (Supplementary Movie 6). We showed snapshots of the aligned tilt series (Supplementary Fig. 7a), and the calculated forward projections by the SIRT (Supplementary Fig. 7b) and the SSR (Supplementary Fig. 7c), respectively. In general, the forward projections show a great agreement with each other and show a structural consistency with respect to the aligned tilt series, which can also be observed from their line profiles (Supplementary Fig. 7d). In contrast to the line profiles of the forward projections by the SIRT and the SSR, a slightly higher overall intensity of the aligned tilt series was observed. This can be ascribed to the presence of a carbon layer induced by the electron beam illumination during the tilt series acquisition (Supplementary Fig. 3). We remark that although SSR is already more advanced than any other reconstruction algorithm for the quantification of the assemblies composed of spherical building blocks, yet it has its limitation like any other type of reconstruction algorithms. The identification of the binary species depends on the gray values from the volume of the SIRT reconstruction. We should note that due to the presence of the background from carbon grid, carbon contamination and the presence of missing wedge which commonly exists in electron tomography experiments, it is possible that some particle were misidentified. As we wrote in the Methods section in the main text, we estimate that around 1% of particle might be misidentified by the SSR reconstructions. We therefore conclude that SSR is a proper algorithm for a quantitative analysis of large-sized assemblies composed of spherical building blocks.

Supplementary Figures

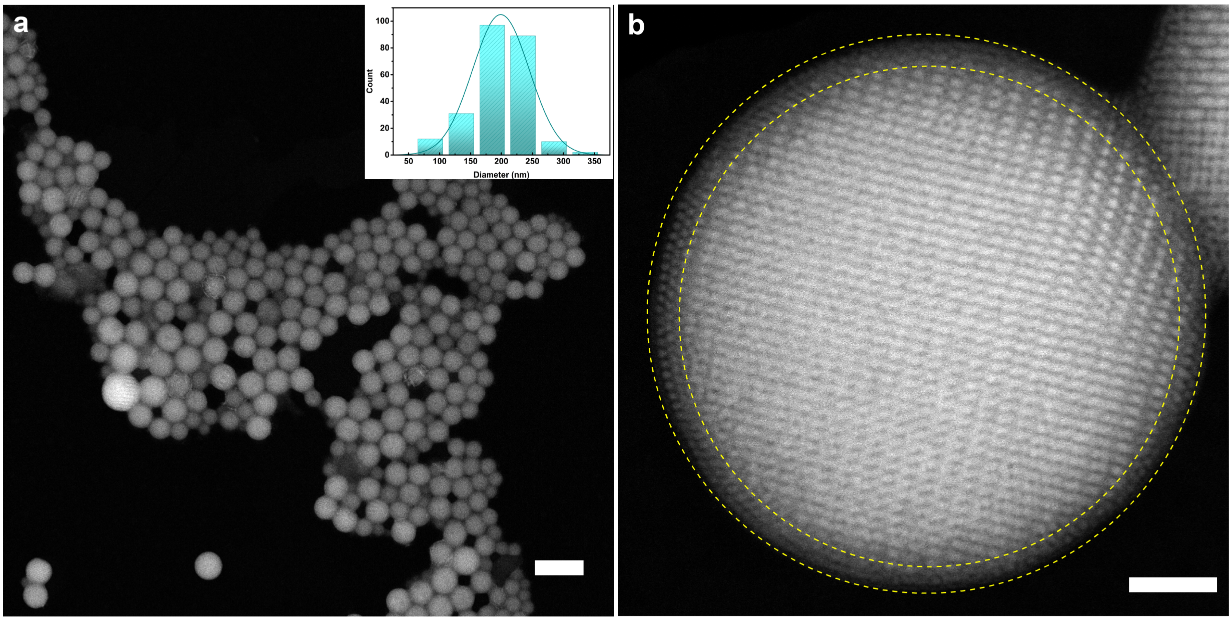

Supplementary Figure 1: Self-assembled Laves supraparticles (SPs) consisting of PbSe NCs and CdSe NCs. a) Overview of self-assembled Laves SPs at low magnification. b) A Laves SP with a diameter of 306 nm showing a core-shell structure. The shell is highlighted between two yellow dashed circle. Inset, size distribution of the SPs. Scale bar, 500 nm (a), 50 nm (b).

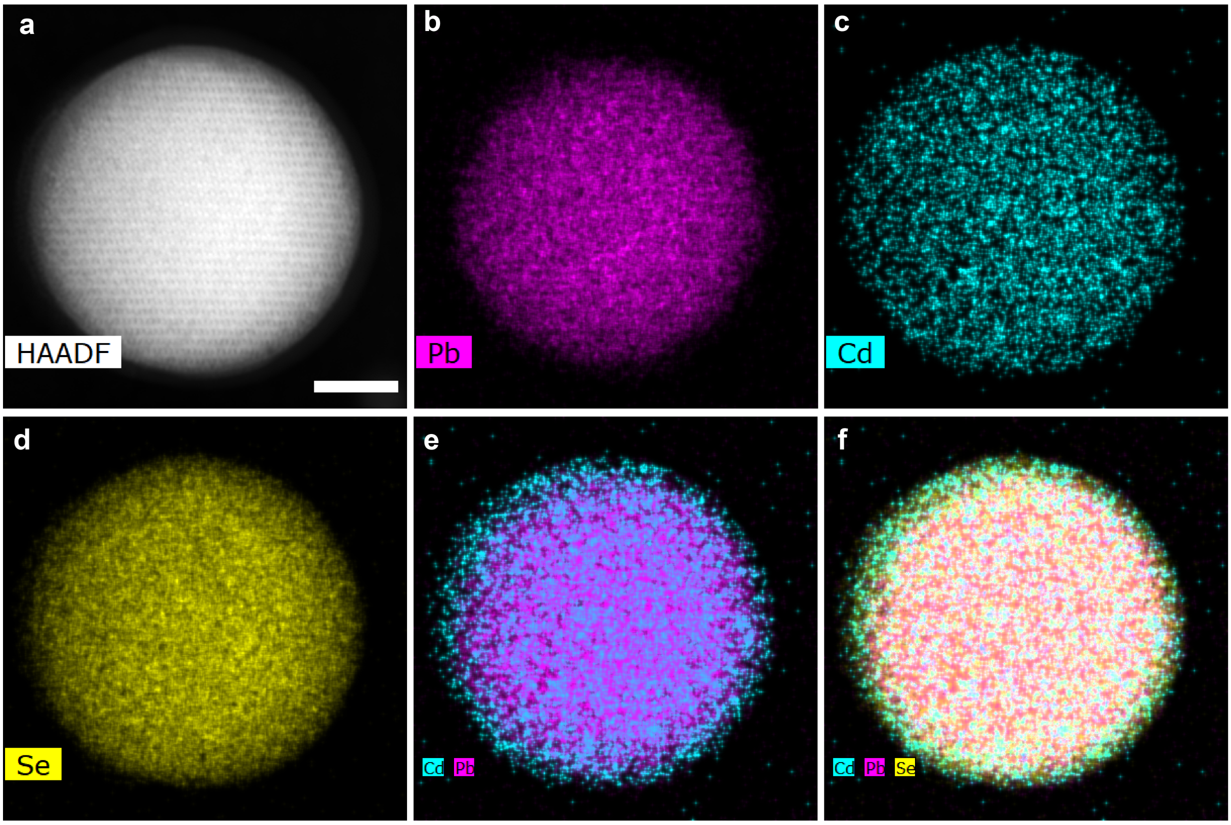

Supplementary Figure 2: Energy dispersive X-Ray spectroscopy chemical mapping of a Laves SP. a) High-angle annular dark-field scanning transmission electron microscopy (HAADF-STEM) image of a Laves SP. Elemental distributions of b) Pb, c) Cd , d) Se, superimposition of e) Cd and Pb and superimposition of f) Cd, Pb and Se. Scale bar, 80 nm.

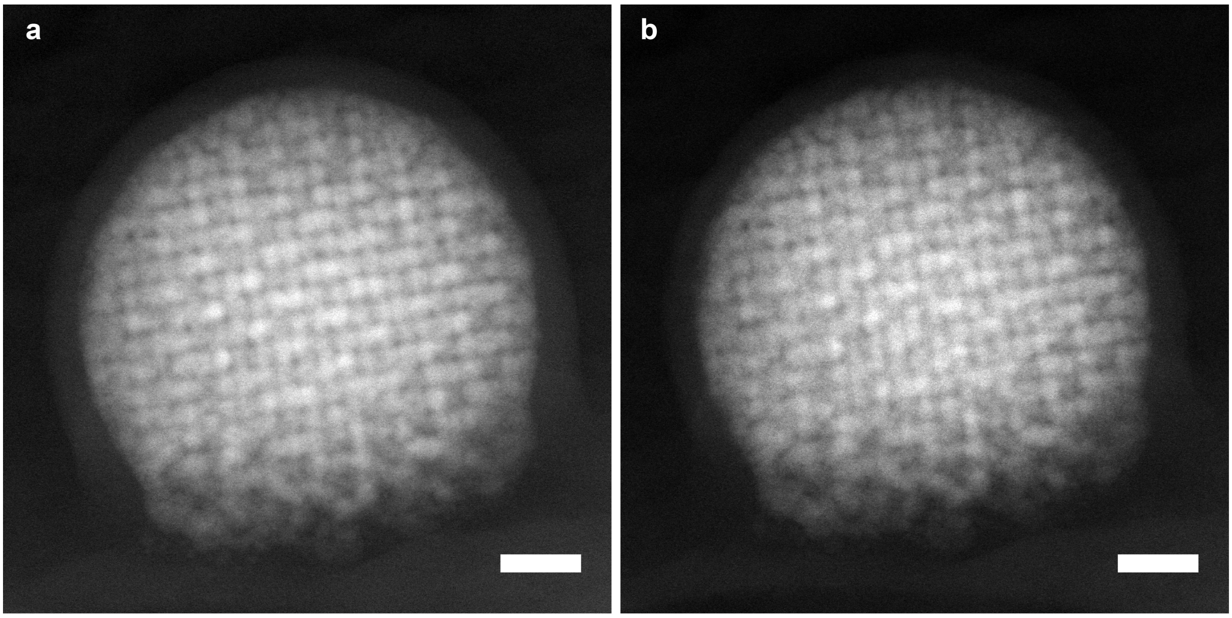

Supplementary Figure 3: Structural consistency of the 115 nm SP. HAADF-STEM images of the 115 nm SP a) before and b) after a tilt series at +66°, showing structural robustness. Scale bars, 20 nm. Grey shell outside the SP represents a carbon layer that transformed from ligands induced by electron beam.

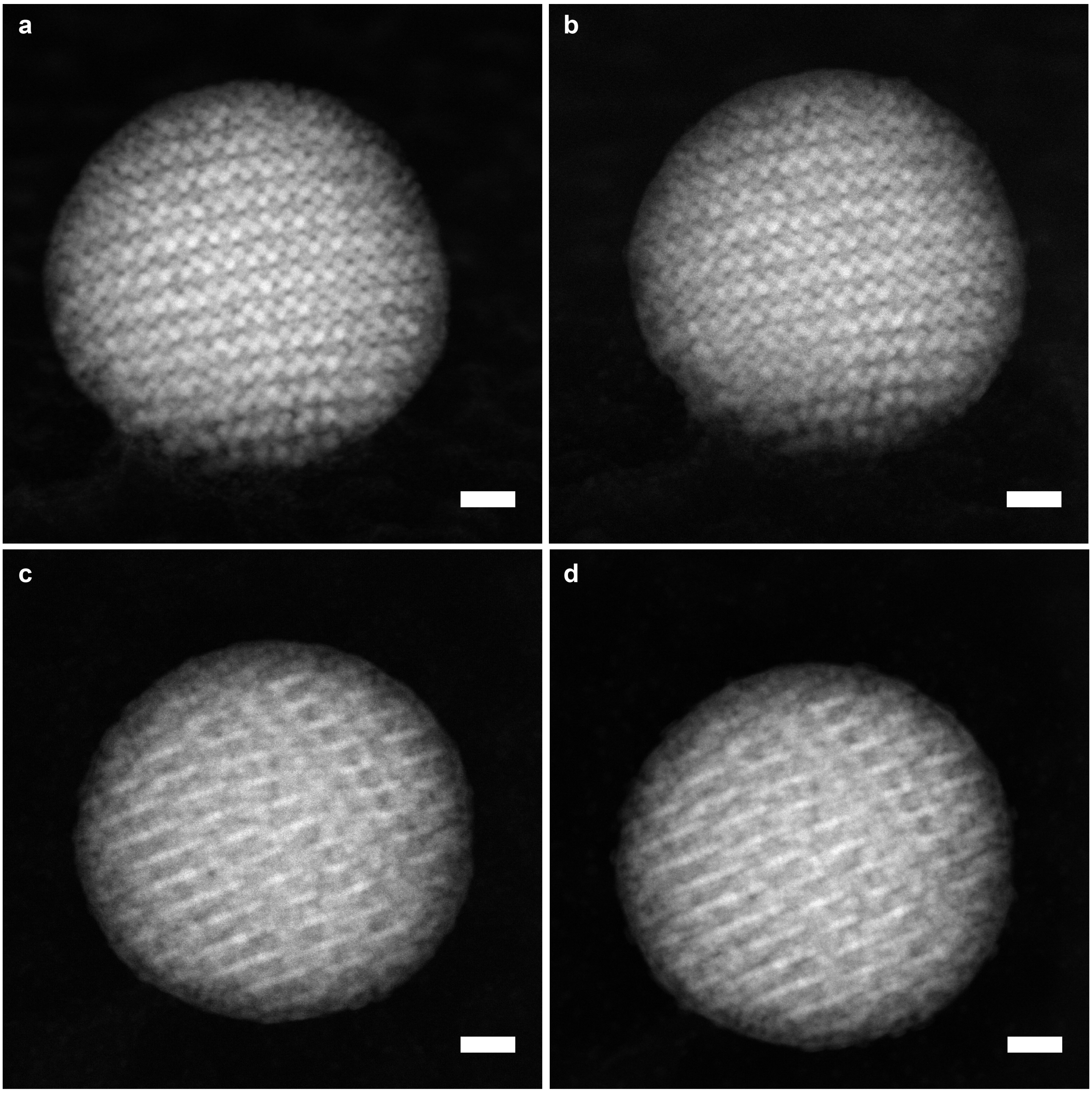

Supplementary Figure 4: Structural consistency of the 150 nm SP. HAADF-STEM images of the 150 nm SP a) before and b) after a tilt series at +62°, c) during and d) after the tilt series at 0°. Scale bars, 20 nm.

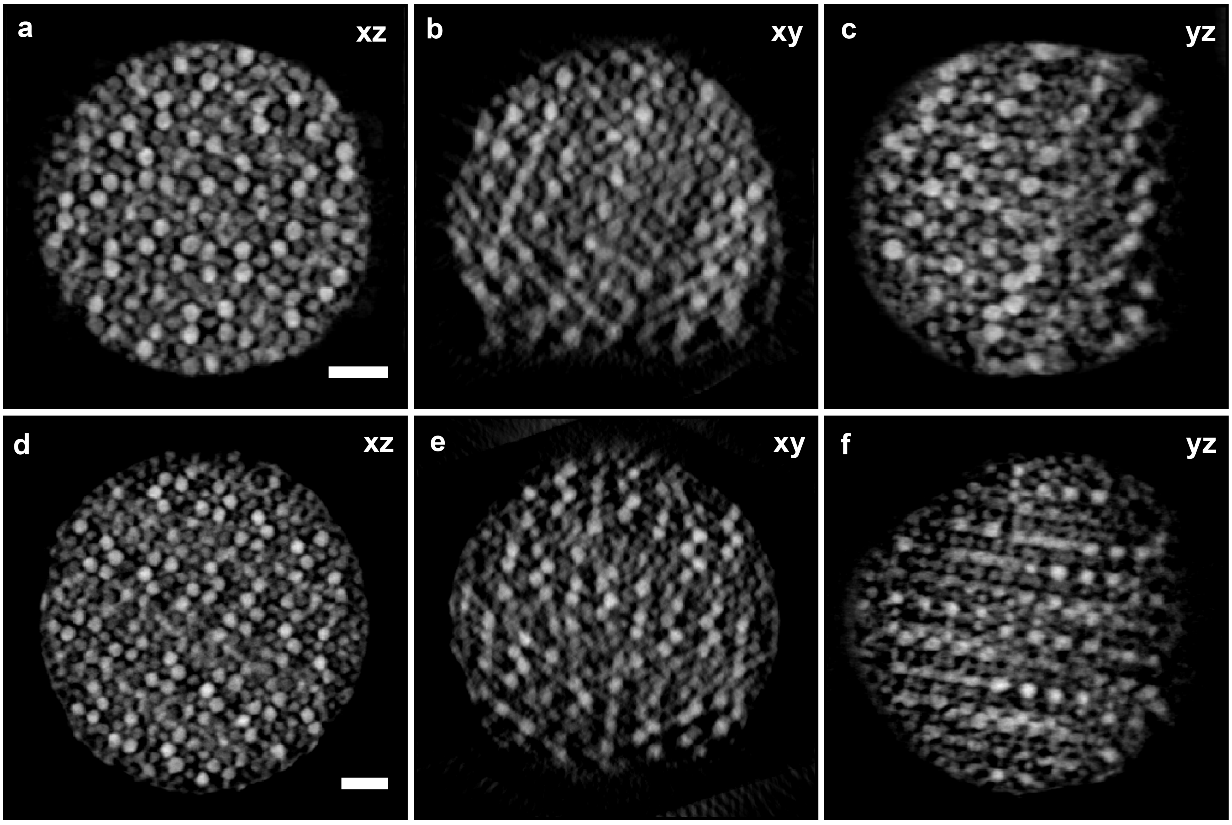

Supplementary Figure 5: Orthoslices of the two SPs viewed from three different directions in this study. Orthoslices of the a-c) 115 nm and d-f) 150 nm SPs viewed along the a,d) y, b,e) z and c,f) x axis, respectively. Scale bars, 20 nm.

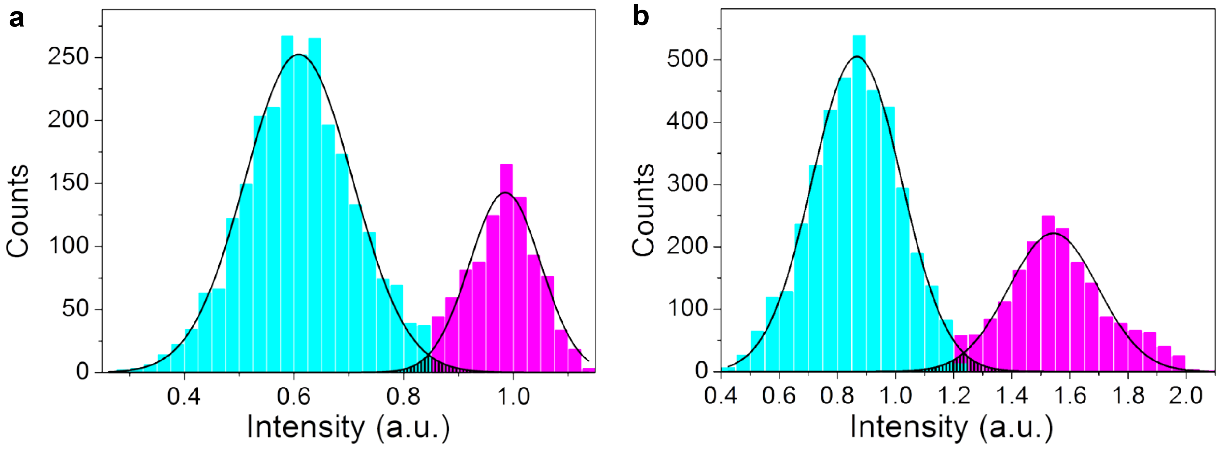

Supplementary Figure 6: Pixel intensity distributions of reconstructed particles. Distribution of the summed pixel intensities of the particles in the a) 115 nm and b) 150 nm sized SPs. The black curves are two Gaussian fitted to the distributions, from which threshold values were determined to differentiate between the CdSe NCs (cyan) and PbSe NCs (magenta). The overlap between the two Gaussian curves gives an estimate for the percentage of particles that might be misidentified ($\sim 1\%$ for both SPs).

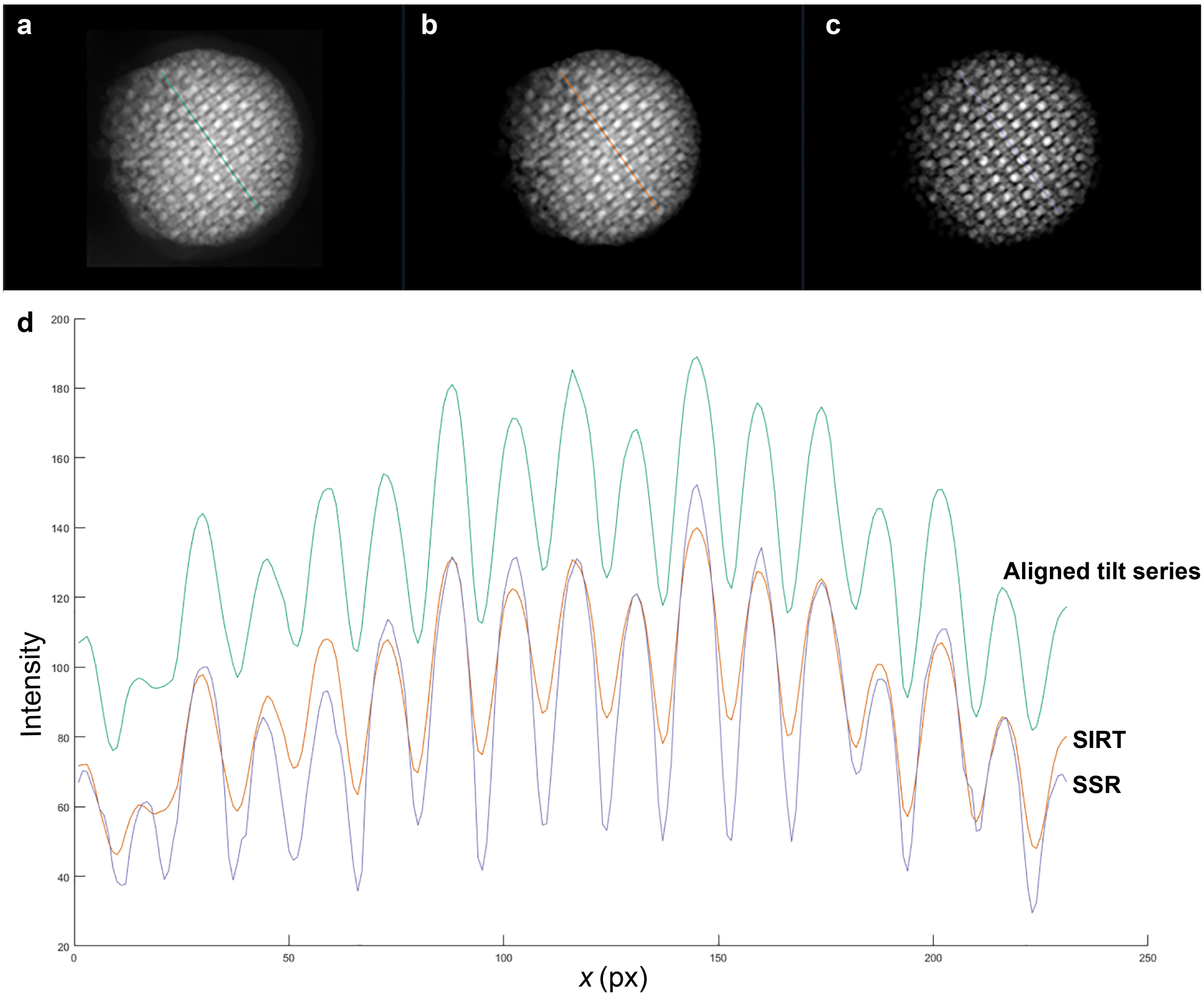

Supplementary Figure 7: Validation of the SSR reconstructions. a-c) Snapshots and d) line profiles of a) the aligned tilt series, calculated forward projections by the b) SIRT and c) SSR algorithms, respectively. The lines in a-c indicate the direction of the profiles shown in d.

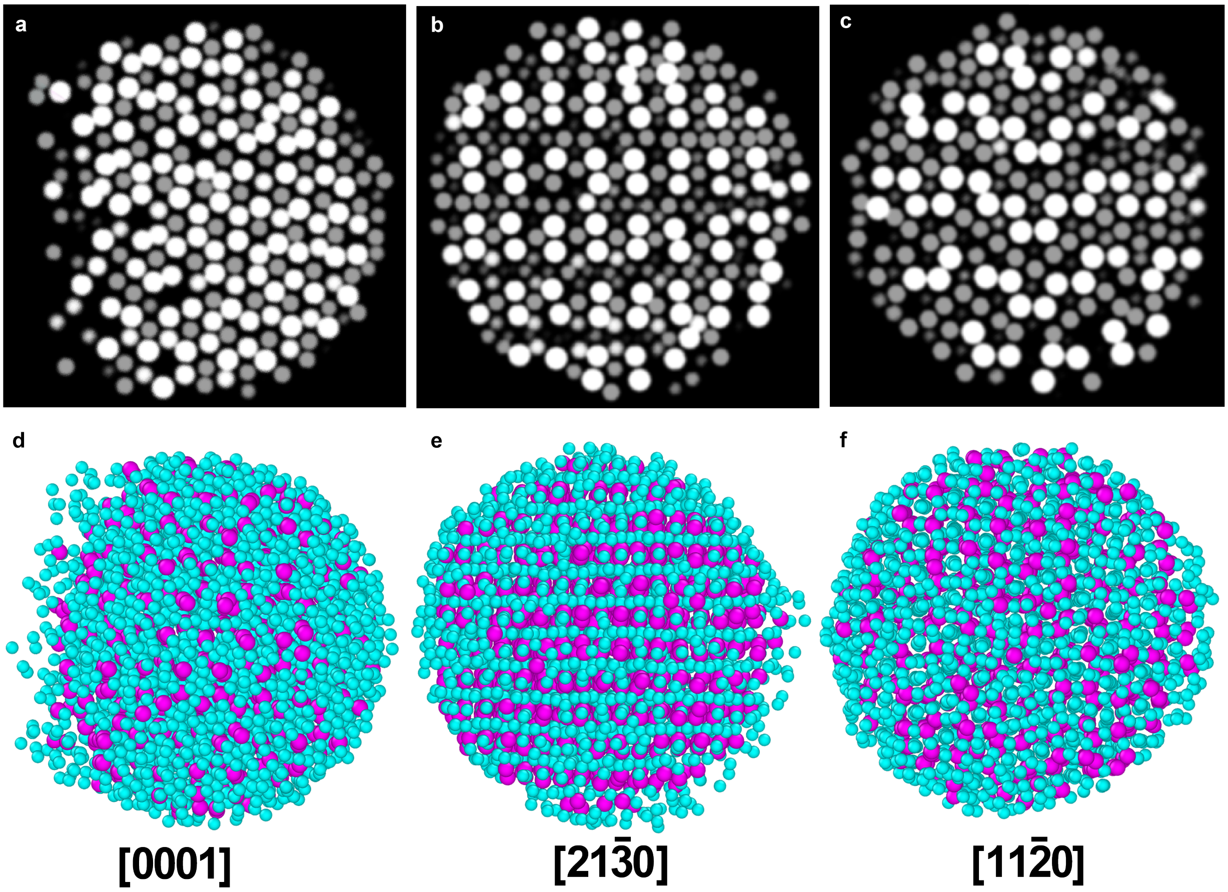

Supplementary Figure 8: Sparse Sphere Reconstruction (SSR) reconstruction of the 115 nm Laves SP. a-c) Orthoslices through the reconstruction and d-f) rendering of the reconstructed volume using SSR algorithm viewed along the a,d) [0001], b,e) [21$\bar{3}$0] and c,f) [11$\bar{2}$0] projections.

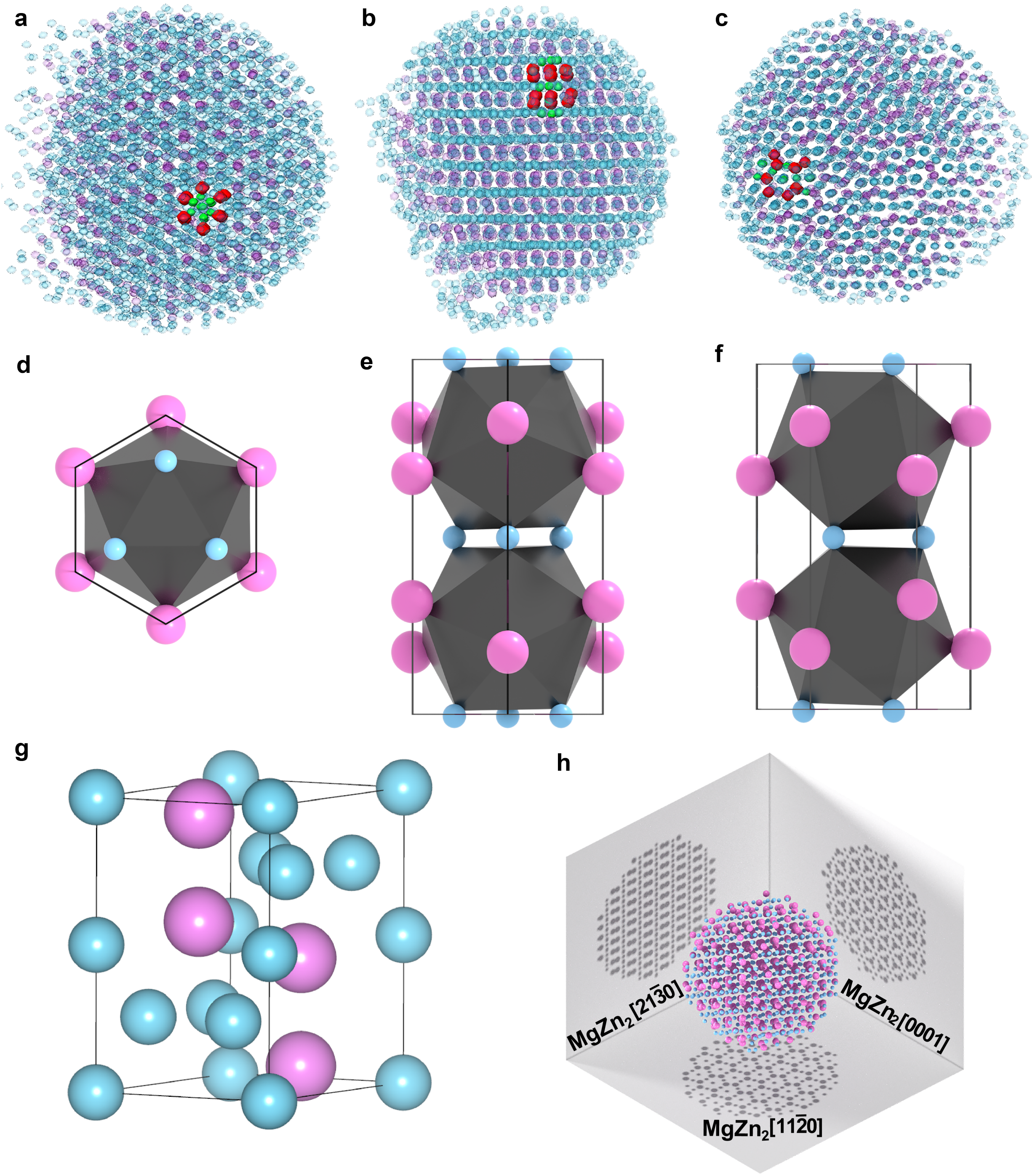

Supplementary Figure 9: Unit cell analysis of the 115 nm SP. a-c) SSR reconstruction of the 115 nm SP, simulated with a reduced particles size enabling an easier manual segmentation viewed along different zone axis. Isolated region of the SP is segmented from the reconstruction for further study of the structure, where the PbSe NCs and CdSe NCs are coloured red and green. d-f) Model built from the coordinates of the manually segmented regions from different zone axis. g) Unit cell confirming the $MgZn_2$ structure. h) A perfect $MgZn_2$ SP built by replicating the $MgZn_2$ unit cell, casting three orthogonal shadows, showing the main zone axis.

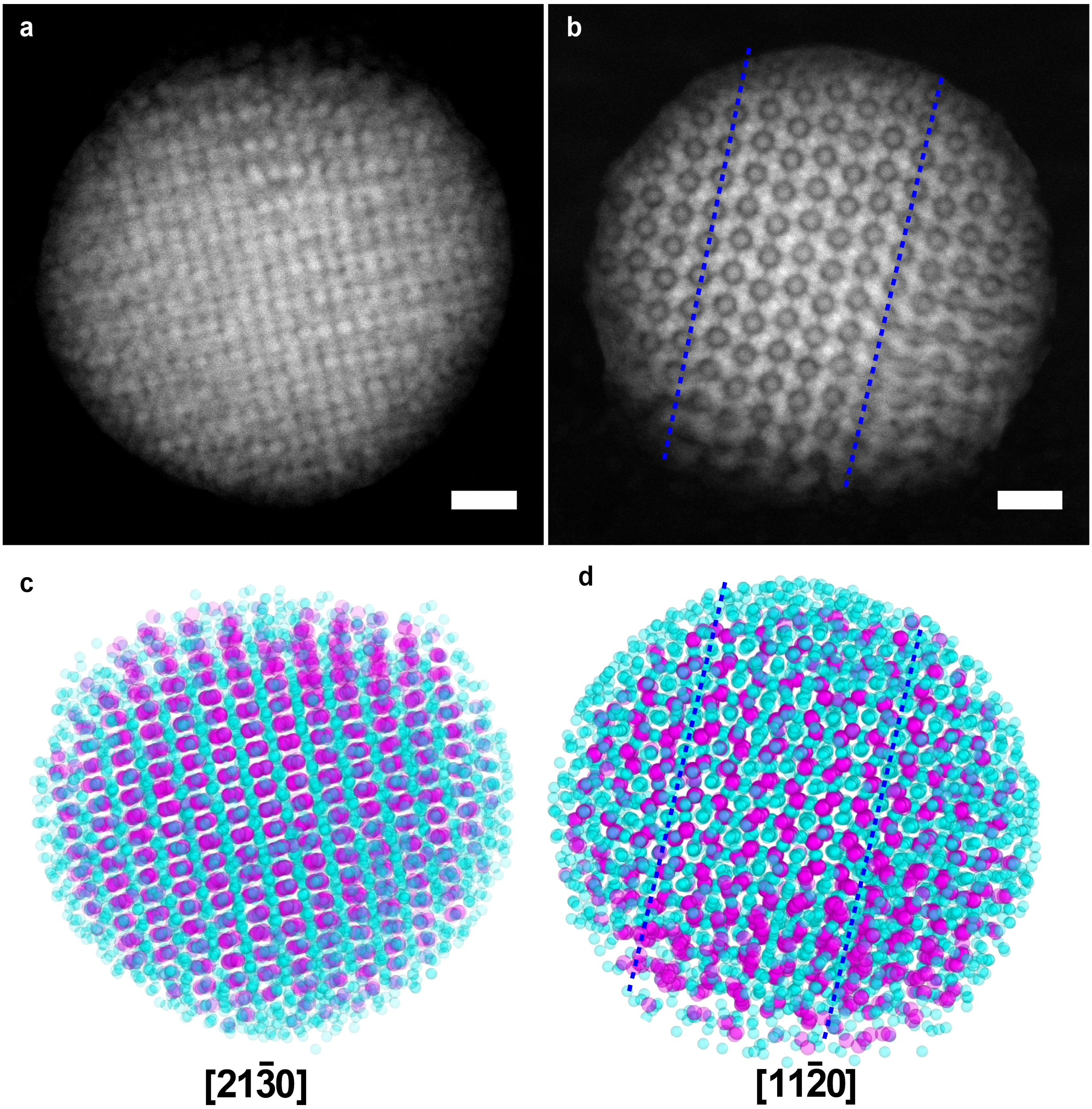

Supplementary Figure 10: A self-assembled Laves SP with a diameter of 150 nm viewed along two zone axis. a-b) HAADF-STEM projection images and c-d) SSR reconstruction renderings of the structure viewed along the a,c) [21$\bar{3}$0] and b,d) [11$\bar{2}$0] projections. Note that transparency of the binary species shown in panel c and d was increased for visual clarity. Stacking faults are marked by dark blue dashed lines. Scale bars, 20 nm.

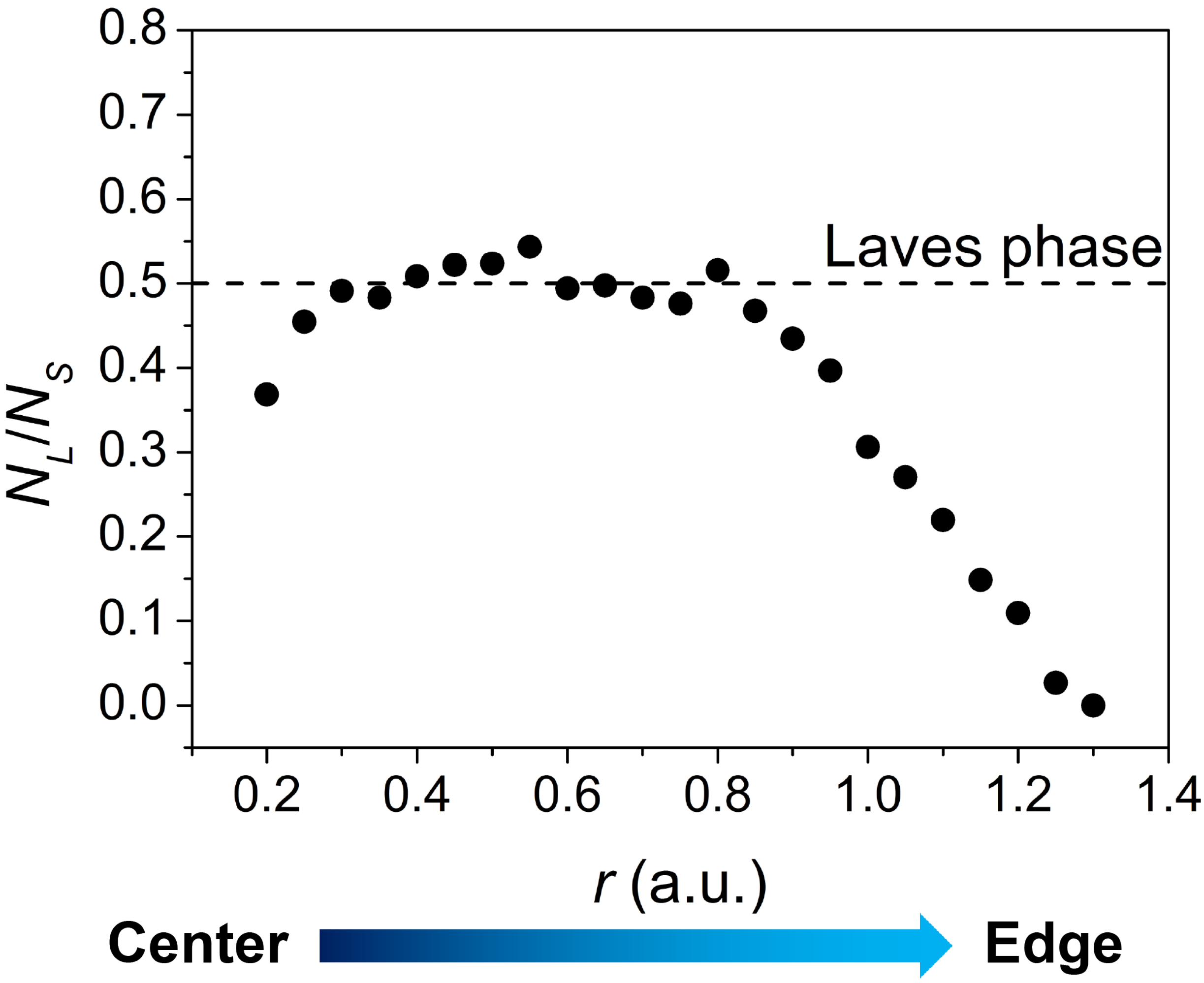

Supplementary Figure 11: Number ratio of L to S species in the 115 nm SP. The number ratio $\frac{N_L}{N_S}$ of L (PbSe NCs) to S (CdSe NCs) species was calculated by drawing a shell with radius r and thickness $d = 0.2$ around the center of mass of the SP. At $r < 0.3$ too few particles were taken into account to get proper statistics, while at $r > 0.8$ the shell reaches outside the Laves crystal, where there is an excess of S species. In the region $0.3 \leq r \leq 0.8$, $\langle \frac{N_L}{N_S} \rangle = 0.50$ (standard deviation is 0.02), in accordance with the Laves phase stoichiometry.

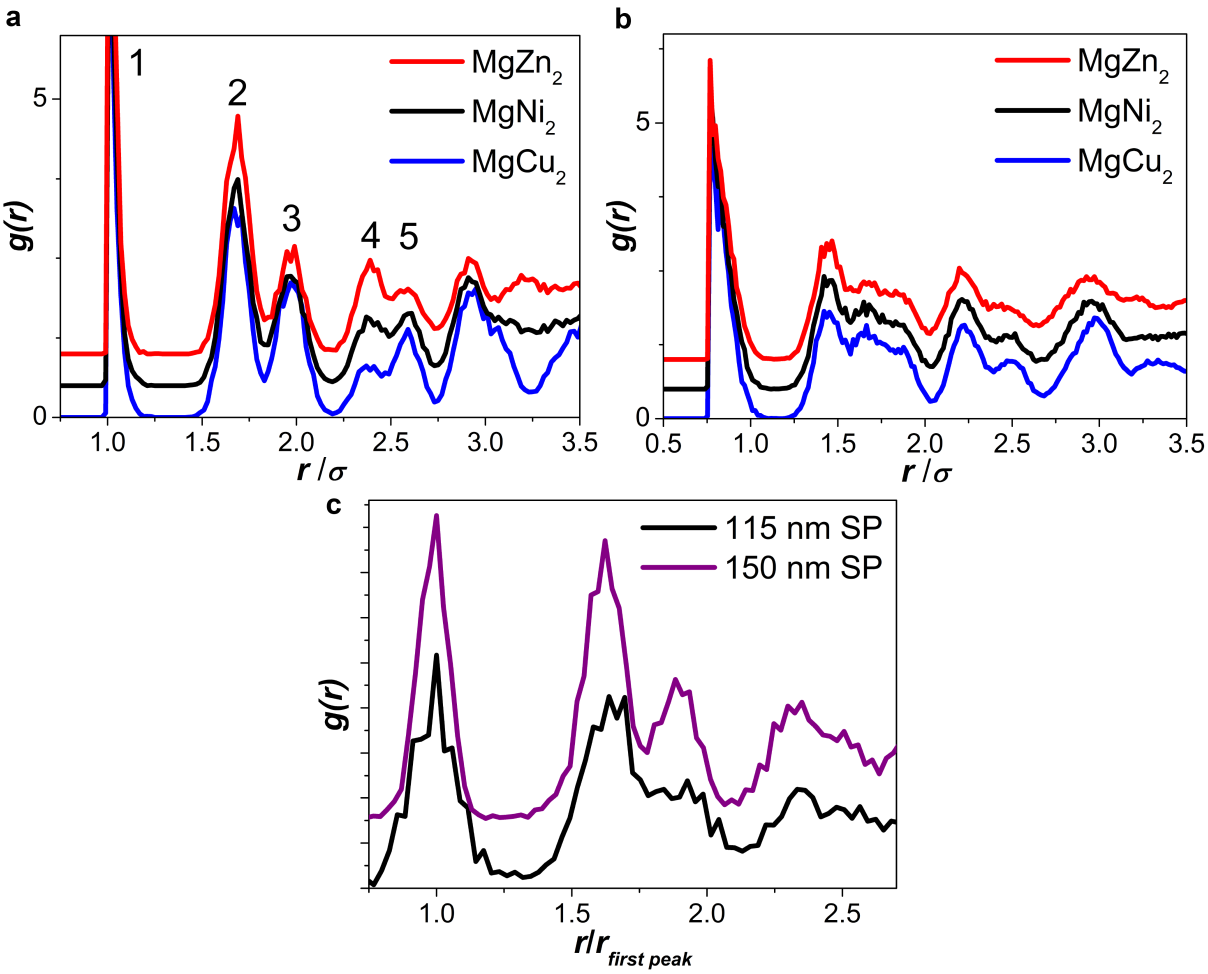

Supplementary Figure 12: Radial distribution function (RDF) of the Laves crystal structures and the SPs. RDF of a) the L and b) S species in the reference equilibrated Laves crystal structures: $MgZn_2$ (red), $MgZn_2$ (black) and $MgCu_2$ (blue). c) RDF of the 115 (black) and 150 (purple) nm sized SPs.

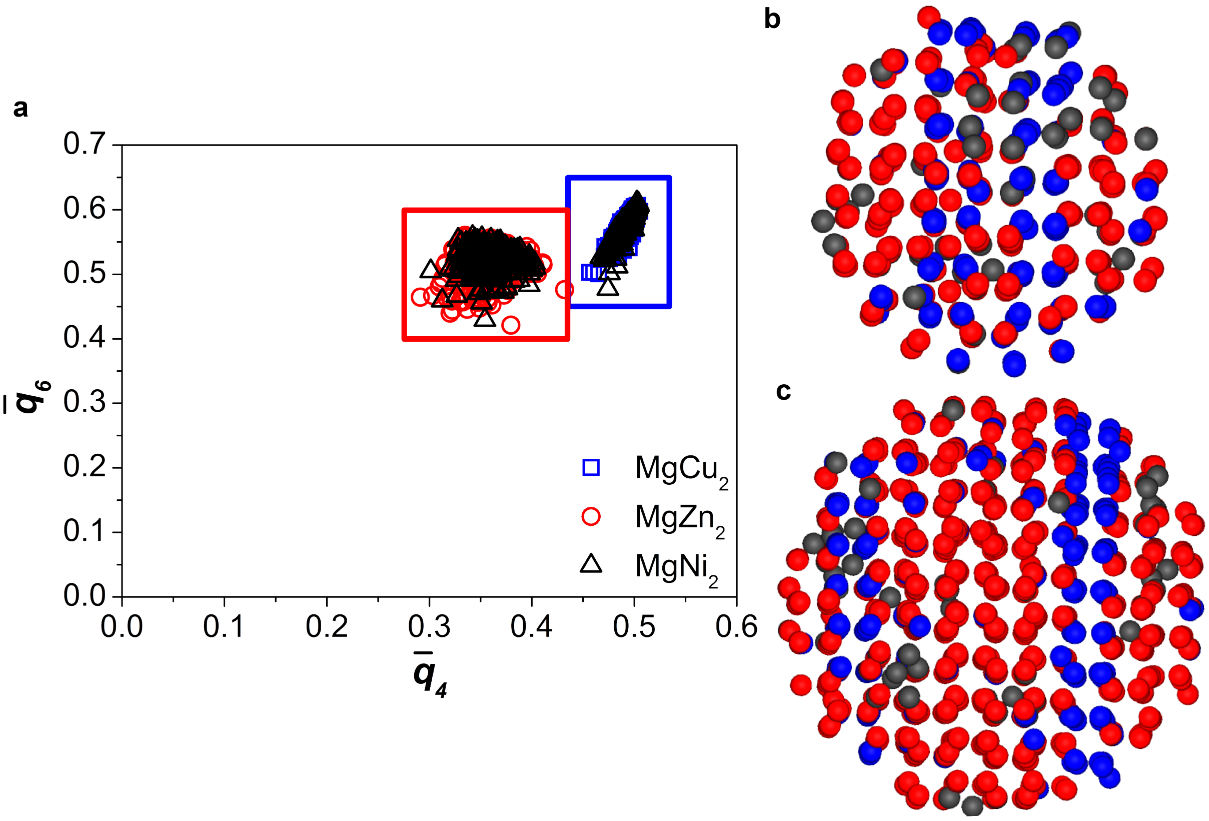

Supplementary Figure 13: Bond-orientational order parameter (BOP) of L species (PbSe NCs). a) Scatter plot of the BOP values of the L species in the simulated Laves phases $MgZn_2$ (red), $MgCu_2$ (blue) and $MgNi_2$ (black). Computer renderings of the L species in the b) 115 nm and c) 150 nm SPs. Particles are coloured to their presence in the rectangles in the BOPs scatter plot (see Fig. 4 in the main text), while particles outside the rectangles are coloured grey.

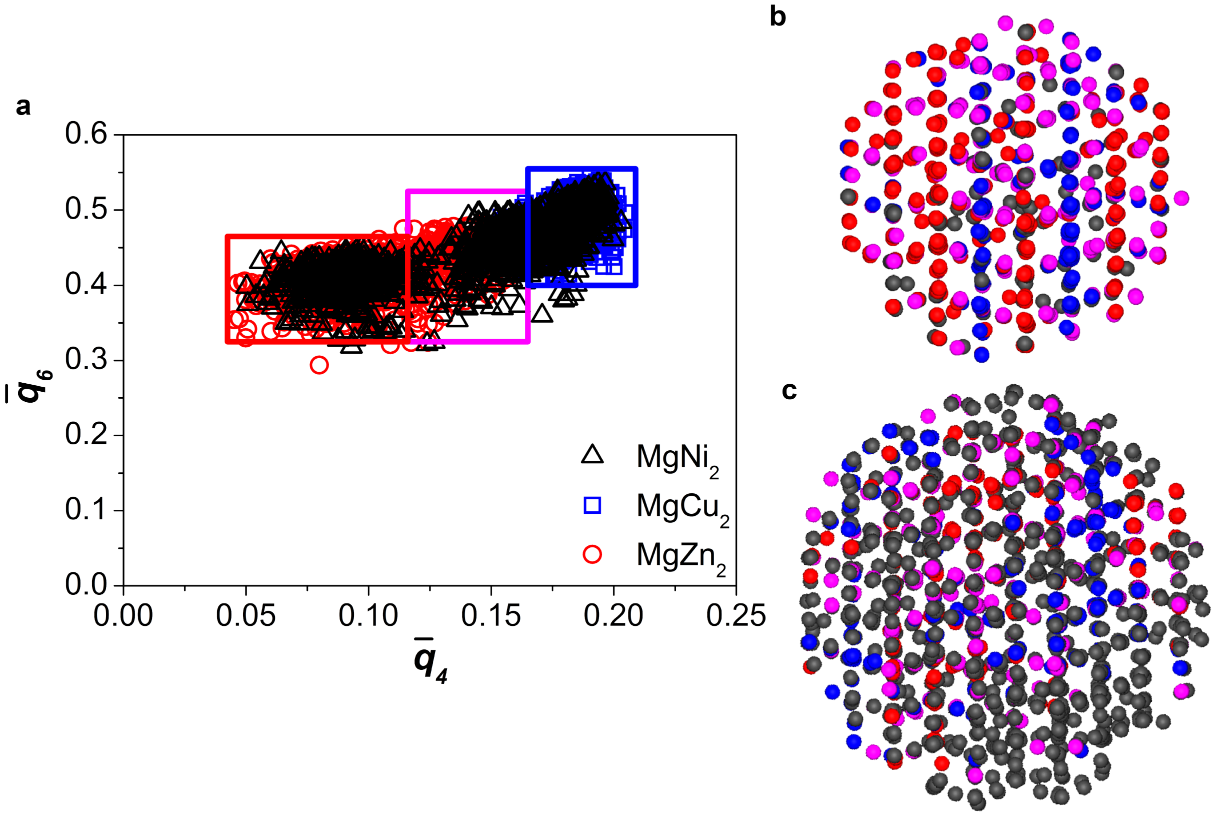

Supplementary Figure 14: BOP analysis of *S* species (CdSe NCs). a) Scatter plot of the BOP values of the *S* species in the simulated Laves phases $MgZn_2$ (red), $MgCu_2$ (blue) and $MgNi_2$ (black). Computer renderings of the *S* species in the b) 115 nm and c) 150 nm SPs. Particles are coloured to their presence in the rectangles in the BOPs scatter plot (see Fig. 5 in the main text), while particles outside the rectangles are coloured grey.

Supplementary References

1. Pietryga, J. M. *et al.* Utilizing the lability of lead selenide to produce heterostructured nanocrystals with bright, stable infrared emission. *J. Am. Chem. Soc.* **130,** 4879–4885 (2008).

2. Steckel, J. S., Yen, B. K. H., Oertel, D. C. & Bawendi, M. G. On the mechanism of lead chalcogenide nanocrystal formation. *J. Am. Chem. Soc.* **128,** 13032–13033 (2006).

3. Goris, B., Van den Broek, W., Batenburg, K. J., Heidari Mezerji, H. & Bals, S. Electron tomography based on a total variation minimization reconstruction technique. *Ultramicroscopy* **113,** 120–130 (2012).

4. Batenburg, K. J. & Sijbers, J. DART: A practical reconstruction algorithm for discrete tomography. *IEEE Trans. Inf. Theory.* **20,** 2542–2553 (2011).

5. Zanaga, D. *et al.* Quantitative 3D analysis of huge nanoparticle assemblies. *Nanoscale* **8,** 292–299 (2016).

6. Wang, D. *et al.* Binary icosahedral clusters of hard spheres in spherical confinement. *Nat. Phys.* **17,** 128–134 (2021).

7. Yang, Z. *et al.* Supracrystalline colloidal eggs: epitaxial growth and free standing three-dimensional supracrystals in nanoscaled colloidosomes. *J. Am. Chem. Soc.* **138,** 3493–3500 (2016).
