# Supplementary material for: Quantitative 3D real-space analysis of Laves phase supraparticles
Source: Nat Commun. 2021 Jun 25;12:3980. doi: 10.1038/s41467-021-24227-0 (PMC8233429; doi:10.1038/s41467-021-24227-0)
Supplement: Supplementary file 2 — Description of Additional Supplementary Files [file 41467_2021_24227_MOESM2_ESM.pdf]

Description of Additional Supplementary Files

File Name: Supplementary Movie 1
Description: A tomography tilt series of a 115 nm Laves supraparticle composed of CdSe and PbSe nanocrystals.

File Name: Supplementary Movie 2
Description: Three-dimensional representation and orthoslices views of the reconstructed 115 nm Laves supraparticle by SSR algorithm. Large PbSe and small CdSe nanocrystals are coloured in magenta and cyan, respectively.

File Name: Supplementary Movie 3
Description: A tomography tilt series of a 150 nm Laves supraparticle composed of small CdSe and large PbSe nanocrystals.

File Name: Supplementary Movie 4
Description: Three-dimensional representation and orthoslices views of the reconstructed 150 nm Laves supraparticle by SSR algorithm. Large PbSe and small CdSe nanocrystals are coloured in magenta and cyan, respectively.

File Name: Supplementary Movie 5
Description: A movie showing computed forward projections of the 115 nm supraparticle by the SIRT algorithm.

File Name: Supplementary Movie 6
Description: A movie showing computed forward projections of the 115 nm supraparticle by the SSR algorithm.

File Name: Supplementary Data 1
Description: Crystal structure of the $MgZn_2$ Laves phase.

File Name: Supplementary Data 2
Description: Crystal structure of the $MgCu_2$ Laves phase.

File Name: Supplementary Data 3
Description: Crystal structure of the $MgNi_2$ Laves phase.

File Name: Supplementary Data 4
Description: A 115 nm binary Laves phase supraparticle composed of 3,432 nanocrystals.

File Name: Supplementary Data 5
Description: A 150 nm binary Laves phase supraparticle composed of 5,762 nanocrystals.

File Name: Supplementary Data 6
Description: Bond order analysis of large species in MgZn2 structure.

File Name: Supplementary Data 7
Description: Bond order analysis of small species in $MgCu_2$ structure.

File Name: Supplementary Data 8
Description: Bond order analysis of large species in $MgNi_2$ structure.

File Name: Supplementary Data 9
Description: Bond order analysis of large nanocrystals in 115 nm supraparticle.

File Name: Supplementary Data 10
Description: Bond order analysis of large nanocrystals in 150 nm supraparticle.

File Name: Supplementary Data 11
Description: Bond order analysis of small species in $MgZn_2$ structure

File Name: Supplementary Data 12
Description: Bond order analysis of small species in $MgCu_2$ structure.

File Name: Supplementary Data 13
Description: Bond order analysis of small species in $MgNi_2$ structure.

File Name: Supplementary Data 14
Description: Bond order analysis of small nanocrystals in 115 nm supraparticle.

File Name: Supplementary Data 15
Description: Bond order analysis of large nanocrystals in 150 nm supraparticle.
