# Supplementary material for: Quantitative 3D real-space analysis of Laves phase supraparticles
Source: Nat Commun. 2021 Jun 25;12:3980. doi: 10.1038/s41467-021-24227-0 (PMC8233429; doi:10.1038/s41467-021-24227-0)
Supplement: Supplementary file 4 — Supplementary Data 2 [file 41467_2021_24227_MOESM4_ESM.html]

MgCu<sub>2</sub> Laves phase


## Supplementary Data 2: MgCu2 Laves phase

Crystal structure of the MgCu2 Laves phase. The slider at the bottom can be used to visualize the inside. Click and drag to rotate.  
  
(b) Toggle the drawing of the simulation box.  
(Shift + Left) Rotate left 90 deg.  
(Left) Rotate left.  
(Shift + Down) Rotate down 90 deg.  
(Down) Rotate down.  
(Shift + Right) Rotate up 90 deg.  
(Right) Rotate right.  
(Shift + Up) Rotate up 90 deg.  
(Up) Rotate up.  
(Space) Stop rotation.  
(-) Zoom out.  
(+) Zoom in.  

Made using  Visual colloids.
