# Supplementary material for: Quantitative 3D real-space analysis of Laves phase supraparticles
Source: Nat Commun. 2021 Jun 25;12:3980. doi: 10.1038/s41467-021-24227-0 (PMC8233429; doi:10.1038/s41467-021-24227-0)
Supplement: Supplementary file 7 — Supplementary Data 5 [file 41467_2021_24227_MOESM7_ESM.html]

Reconstruction of a 150 nm supraparticle


## Supplementary Data 5: Reconstruction of a 150 nm supraparticle

Reconstruction of a 150 nm supraparticle containing large (magenta) and small (cyan) species. The size of the particles is reduced to increase visibility.

Made using  Visual colloids.
