# Supplementary material for: Quantitative 3D real-space analysis of Laves phase supraparticles
Source: Nat Commun. 2021 Jun 25;12:3980. doi: 10.1038/s41467-021-24227-0 (PMC8233429; doi:10.1038/s41467-021-24227-0)
Supplement: Supplementary file 8 — Supplementary Data 6 [file 41467_2021_24227_MOESM8_ESM.html]

Bond order analysis of large species in MgZn<sub>2</sub> structure


## Supplementary Data 6: Bond order analysis of large species in MgZn2 structure

Large species of an equilibrated MgZn2 structure. Particles are coloured according their bond order parameter values (see Supplmenetary Fig. 13a).

Made using  Visual colloids.
