# Supplementary material for: Quantitative 3D real-space analysis of Laves phase supraparticles
Source: Nat Commun. 2021 Jun 25;12:3980. doi: 10.1038/s41467-021-24227-0 (PMC8233429; doi:10.1038/s41467-021-24227-0)
Supplement: Supplementary file 9 — Supplementary Data 7 [file 41467_2021_24227_MOESM9_ESM.html]

Bond order analysis of large species in MgCu<sub>2</sub> structure


## Supplementary Data 7: Bond order analysis of large species in MgCu2 structure

Large species of an equilibrated MgCu2 structure. Particles are coloured according their bond order parameter values (see Supplementary Fig. 13a).

Made using  Visual colloids.
