# Supplementary material for: Quantitative 3D real-space analysis of Laves phase supraparticles
Source: Nat Commun. 2021 Jun 25;12:3980. doi: 10.1038/s41467-021-24227-0 (PMC8233429; doi:10.1038/s41467-021-24227-0)
Supplement: Supplementary file 10 — Supplementary Data 8 [file 41467_2021_24227_MOESM10_ESM.html]

Bond order analysis of large species in MgNi<sub>2</sub> structure


## Supplementary Data 8: Bond order analysis of large species in MgNi2 structure

Large species of an equilibrated MgNi2 structure. Particles are coloured according their bond order parameter values (see Supplementary Fig. 13a).

Made using  Visual colloids.
