# Supplementary material for: Quantitative 3D real-space analysis of Laves phase supraparticles
Source: Nat Commun. 2021 Jun 25;12:3980. doi: 10.1038/s41467-021-24227-0 (PMC8233429; doi:10.1038/s41467-021-24227-0)
Supplement: Supplementary file 11 — Supplementary Data 9 [file 41467_2021_24227_MOESM11_ESM.html]

Bond order analysis of large NCs in 115 nm supraparticle


## Supplementary Data 9: Bond order analysis of large nanocrystals in 115 nm supraparticle

Large nanocrystals in 115 nm supraparticle. The particles are coloured according their bond order parameter values (see also Fig. 4d). Particles outside the red or blue boxes in panel d are left out in the rendering.

Made using  Visual colloids.
