# Supplementary material for: Quantitative 3D real-space analysis of Laves phase supraparticles
Source: Nat Commun. 2021 Jun 25;12:3980. doi: 10.1038/s41467-021-24227-0 (PMC8233429; doi:10.1038/s41467-021-24227-0)
Supplement: Supplementary file 13 — Supplementary Data 11 [file 41467_2021_24227_MOESM13_ESM.html]

Bond order analysis of small species in MgZn<sub>2</sub> structure


## Supplementary Data 11: Bond order analysis of small species in MgZn2 structure

Small species of an equilibrated MgZn2 structure. Particles are coloured according their bond order parameter values (see Supplementary Fig. 14a).

Made using  Visual colloids.
