# Supplementary material for: Quantitative 3D real-space analysis of Laves phase supraparticles
Source: Nat Commun. 2021 Jun 25;12:3980. doi: 10.1038/s41467-021-24227-0 (PMC8233429; doi:10.1038/s41467-021-24227-0)
Supplement: Supplementary file 14 — Supplementary Data 12 [file 41467_2021_24227_MOESM14_ESM.html]

Bond order analysis of small species in MgCu<sub>2</sub> structure


## Supplementary Data 12: Bond order analysis of small species in MgCu2 structure

Small species of an equilibrated MgCu2 structure. Particles are coloured according their bond order parameter values (see Supplementary Fig. 14a).

Made using  Visual colloids.
