# Supplementary material for: Quantitative 3D real-space analysis of Laves phase supraparticles
Source: Nat Commun. 2021 Jun 25;12:3980. doi: 10.1038/s41467-021-24227-0 (PMC8233429; doi:10.1038/s41467-021-24227-0)
Supplement: Supplementary file 15 — Supplementary Data 13 [file 41467_2021_24227_MOESM15_ESM.html]

Bond order analysis of small species in MgNi<sub>2</sub> structure


## Supplementary Data 13: Bond order analysis of small species in MgNi2 structure

Small species of an equilibrated MgNi2 structure. Particles are coloured according their bond order parameter values (see Supplementary Fig. 14a).

Made using  Visual colloids.
