# Supplementary material for: Quantitative 3D real-space analysis of Laves phase supraparticles
Source: Nat Commun. 2021 Jun 25;12:3980. doi: 10.1038/s41467-021-24227-0 (PMC8233429; doi:10.1038/s41467-021-24227-0)
Supplement: Supplementary file 16 — Supplementary Data 14 [file 41467_2021_24227_MOESM16_ESM.html]

Bond order analysis of small NCs in 115 nm supraparticle


## Supplementary Data 14: Bond order analysis of small nanocrystals in 115 nm supraparticle

Small nanocrystals in 115 nm supraparticle. The particles are coloured according their bond order parameter values (see also Fig. 5d). Particles outside the red, magenta or blue boxes in panel d are left out in the rendering.

Made using  Visual colloids.
