# Supplementary material for: Quantitative 3D real-space analysis of Laves phase supraparticles
Source: Nat Commun. 2021 Jun 25;12:3980. doi: 10.1038/s41467-021-24227-0 (PMC8233429; doi:10.1038/s41467-021-24227-0)
Supplement: Supplementary file 17 — Supplementary Data 15 [file 41467_2021_24227_MOESM17_ESM.html]

Bond order analysis of small NCs in 150 nm supraparticle


## Supplementary Data 15: Bond order analysis of small nanocrystals in 150 nm supraparticle

Small nanocrystals in 150 nm supraparticle. The particles are coloured according their bond order parameter values (see also Fig. 5f). Particles outside the red, magenta or blue boxes in panel f are left out in the rendering.

Made using  Visual colloids.
